# Left-censored recurrent event analysis in epidemiological studies: a proposal for when the number of previous episodes is unknown

**DOI:** 10.1186/s12874-022-01503-1

**Published:** 2022-01-16

**Authors:** Gilma Hernández-Herrera, David Moriña, Albert Navarro

**Affiliations:** 1 Instituto de Investigaciones Médicas, Facultad de Medicina, Universidad de Antioquia, Medellín, Colombia; 2 Methodology of Biomedical Research and Public Health, Autonomous University of Barcelona, Cerdanyola del Vallès, Spain; 3 Department of Econometrics, Statistics and Applied Economics, Riskcenter-IREA, University of Barcelona (UB), Barcelona, Spain; 4 Centre de Recerca Matemàtica (CRM), Cerdanyola del Vallès, Spain; 5 Facultat d’Economia i Empresa, Universitat de Barcelona (UB), Avinguda Diagonal, 690-694, 08034 Barcelona, Spain; 6 Psychosocial Risks, Organization of Work and Health (POWAH), Autonomous University of Barcelona (UAB), Cerdanyola del Vallès, Spain; 7 Biostatistics Unit, Faculty of Medicine, Autonomous University of Barcelona (UAB), Cerdanyola del Vallès, Spain

## Abstract

**Background:**

When dealing with recurrent events in observational studies it is common to include subjects who became at risk before follow-up. This phenomenon is known as left censoring, and simply ignoring these prior episodes can lead to biased and inefficient estimates. We aimed to propose a statistical method that performs well in this setting.

**Methods:**

Our proposal was based on the use of models with specific baseline hazards. In this, the number of prior episodes were imputed when unknown and stratified according to whether the subject had been at risk of presenting the event before t = 0. A frailty term was also used. Two formulations were used for this “Specific Hazard Frailty Model Imputed” based on the “counting process” and “gap time.” Performance was then examined in different scenarios through a comprehensive simulation study.

**Results:**

The proposed method performed well even when the percentage of subjects at risk before follow-up was very high. Biases were often below 10% and coverages were around 95%, being somewhat conservative. The gap time approach performed better with constant baseline hazards, whereas the counting process performed better with non-constant baseline hazards.

**Conclusions:**

The use of common baseline methods is not advised when knowledge of prior episodes experienced by a participant is lacking. The approach in this study performed acceptably in most scenarios in which it was evaluated and should be considered an alternative in this context. It has been made freely available to interested researchers as R package *miRecSurv*.

**Supplementary Information:**

The online version contains supplementary material available at 10.1186/s12874-022-01503-1.

## Background

In epidemiological cohort studies, participants may have been at risk of the event of interest before entering follow-up, especially in observational designs. This can lead to unawareness of their history, specifically the time at risk, and whether they have experienced the event (including how often) at study inception, which is problematic when the baseline hazard of the event is time dependent. There are two relevant situations to consider. First, if an event can only occur once and has already happened, the result is already determined and will require specific statistical techniques for analysis. Second, if the outcome of interest is a recurrent event that can occur more than once, the number of prior episodes will be unknown despite the potential for new events. This represents left censoring where the censored variable is of the discrete type and for which we can define different baseline hazards.

There is a need to clarify how data are handled when the prior history is unknown in a cohort and the outcome of interest is a recurrent event with event dependence. Specifically, this concerns situations where we know the moment from when individuals become at risk, but we do now know the number of prior episodes. For example, concerning the risk of sick leave in a work force, we will likely know the start date for employment; however, especially for people with ample trajectory, we may not know how many sick leaves they had prior to this employment. Another example can be seen in cohorts where the outcome is the incidence of infection with the human papilloma virus in adult women. It would be relatively simple to know how long they have been at risk (first sexual intercourse), but we will not know the true number of infections because they are typically asymptomatic.

This study has two objectives. The first is to describe an analysis proposal for recurrent phenomena in the presence of event dependence when the prior history is unknown for some or all subjects. The second is to compare the performance of our proposed model with another that ignores event dependence.

## Methods

### Event dependence

When analyzing a recurrent event, we often observe a phenomenon called event dependency in which the baseline hazard of an episode depends on the number of episodes that have already occurred. To date, the phenomenon has been estimated for falls [1], sickness absence [2, 3], hospitalizations in heart failure [4], and cardiovascular readmission after percutaneous coronary intervention [5]. All of these have shown that the baseline hazard increased most significantly with the number of prior episodes.

Recurrent event analyses in epidemiological studies are well described [6–9], typically as extensions of Cox’s classical model of proportional hazards [10, 11]. Specifically, baseline hazard models, also known as conditional models or Prentice, Williams, and Peterson (PWP) models [12], are used to consider the existence of event dependence. These models assume that the baseline hazard of an episode differs as a function of episodes that have already occurred, stratifying by how many there have been. This allows general or specific effects to be calculated for each episode, with all at-risk individuals included in the first strata, but only those with an episode in the previous strata subsequently considered at risk.

PWP models can be formulated in two ways depending on the risk interval used (i.e., how time is considered) [6]. In the first, called “counting process,” time is handled typically for the survival analysis, always referenced from the start of follow-up such that the beginning of the *k*_*th*_ episode is always posterior at the end of the *k-1*_*st*_. The hazard function is shown below (Eq. 1). In the second format, called “gap time,” time is always considered in relation to the prior episode such that the start of each new episode for a given individual is set at zero (Eq. 2)

1 $${\uplambda}_{\mathrm{i}\mathrm{k}}\left(\mathrm{t}\right)={\uplambda}_{0\mathrm{k}}\left(\mathrm{t}\right){\mathrm{e}}^{{\mathrm{X}}_{\mathrm{i}}\upbeta}{h}_{ik}(t)={h}_{0k}(t){e}^{X_i\beta }$$

2 $${h}_{ik}(t)={h}_{0k}\left(t-{t}_{k-1}\right){e}^{X_i\beta }$$

where *Xi* represents the vector of covariables, *β* represents the coefficients of regression, *k* is the *k*_*th*_ event episode for individual *i*, and *h0 k(t)* is the function of the baseline hazard dependent on *k*.

If the phenomenon under study lacks event dependence, these models can be simplified to a function of the common baseline hazard. These are known as Andersen–Gill models [13] and assign the same baseline hazard independent of the episodes that have already been experienced (Eq. 3).

3 $${h}_i(t)={h}_0(t){e}^{X_i\beta }$$

where *h0(t)* is the common baseline hazard.

### Individual heterogeneity

If a model is perfectly specified, with all possible covariates accounted for, then the baseline hazard reflects the randomness of the event time given the value of the covariates. In practice, however, it is rarely possible to account for all relevant covariates [14]. This requires another aspect to be considered: the unmeasured effects produced by between-subject variability, presumably due to unobserved exposures. This phenomenon is called individual heterogeneity and in practice is analyzed by adding a frailty to the model, *νi*, making an individual random effect to account for the “extra” variability. Given that *νi* has a multiplicative effect, we can imagine that it represents the cumulative effect of one or more omitted covariates [15]. The most commonly adopted frailty terms are *E [νi] = 1* and *V [νi] = θ* [16–18]. Thus, the models specified in Eq. 1, Eq. 2, and Eq. 3 change to those specified in Eq. 4 for “Conditional Frailty Model - Counting Process,” Eq. 5 for “Conditional Frailty Model - Gap Time,” and Eq. 6 for “Shared Frailty Model,” respectively.

4 $${h}_{ik}(t)={\nu}_i{h}_{0k}(t){e}^{X_i\beta }$$

5 $${h}_{ik}(t)={\nu}_i{h}_{0k}\left(t-{t}_{k-1}\right){e}^{X_i\beta }$$

6 $${h}_i(t)={\nu}_i{h}_0(t){e}^{X_i\beta }$$

### The problem of being unaware of the previous history for some individuals

Specific baseline hazard methods, either with or without frailties, can be applied when all required information is known, particularly the number of prior episodes for each individual. In practice, however, this information is not always available, preventing the inclusion of basic information needed to consider event dependence.

**Fig. 1:**
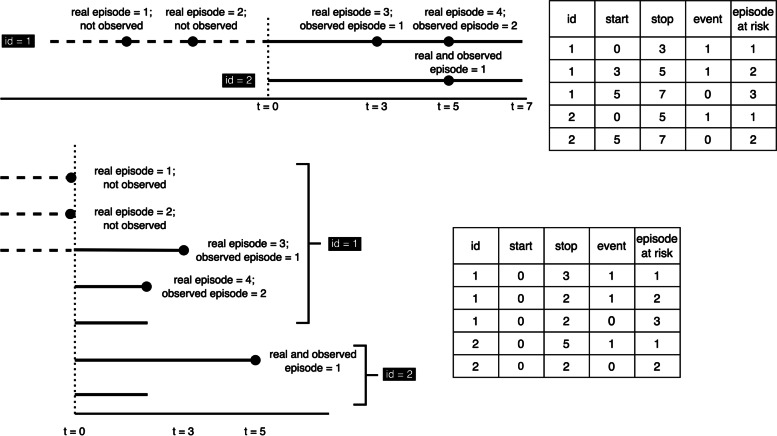
Example history of two individuals presented according to the counting process and gap time formulation. The counting process is shown in the top image and table, and the gap time approach is shown below for two patients (id 1, 2) at time points (t) 0, 3, 5, and 7

Figure 1 shows representative data for two subjects with their respective risk intervals. These are shown according to the counting process on the top and according to gap time on the bottom. Notice that the difference between these formulations is that the gap time “restarts” time to risk at *t = 0* after each episode. When starting our study, the first subject (*id = 1*) had two prior episodes and had been at risk for a considerable amount of time, making this a left-censored observation. However, we are only aware of the two episodes that occur from the start of follow-up. By contrast, for the second subject (*id = 2*), exposure starts at *t = 0*, they have an episode at *t = 5,* and follow-up stops at *t = 7.* The data tables show the resulting analysis: note that the prior history for Subject 1 “disappears” in both approaches such that the risk only appears at the same instant as it does for Subject 2.

Thus, two problems exist. First, if there is event dependence, and we fail to stratify by number of episodes, we will mix individuals that have different baseline hazards at that instant (e.g., risk starts for Subject 2 at baseline, when Subject 1 is already at risk of a third). Second, we mix temporal scales: on one side there will be subjects (e.g., Subject 2) whose follow-up time corresponds with their time at risk, but on the other side there will be subjects (e.g., Subject 1) whose follow-up time does not correspond with their time at risk. Consequently, two subpopulations with different baseline hazards are mixed, which goes against the assumptions of the Cox model and its extensions for recurrent phenomena.

When there is missing data for prior history and specific baseline hazard models are unsuitable, an alternative approach is to ignore that history and fit the models based on common baseline hazards (i.e., eqs. 3 and 6). However, these assign the same baseline hazard to all episodes, and as such, they consider neither the possible effect of the number of prior episodes nor the effect of comparing two subjects at the same instant who have times to risk that can be radically different. The use of such models with common baseline hazards for analyzing recurrent phenomena with event dependence has been shown to be highly inefficient, generating high levels of bias in parameter estimates and confidence intervals. Results can be unsound, even if when the event dependence is of small intensity [19]. Thus, alternative analytic methods are necessary for this context.

### Proposal

Our proposal starts with the assumption that, even though the history of a given subject may be unknown, we do know who was at risk before starting follow-up. This is based on three fundamental considerations: 1) imputing *k*, the number of previous episodes for those subjects at risk before follow-up starts; 2) treating the subpopulation “previously at risk” separately from that “not previously at risk” and 3) using a frailty term to capture the impact of unobserved effects, including uncertainty related to the imputation process*.* This gives two formulations that we call “Specific Hazard Frailty Model Imputed,” as shown in Eq. 7 for the counting process (SPECIFIC.CP) and Eq. 8 for gap time (SPECIFIC.GT).

7 $${h}_{ikr}(t)={\nu}_i{h}_{0 kr}(t){e}^{X_i\beta }$$

8 $${h}_{ikr}(t)={\nu}_i{h}_{0 kr}\left(t-{t}_{k-1}\right){e}^{X_i\beta }$$

where *k* is the number of previous episodes in individual *i* when they are known or the imputed value when they are not known; *r* indicates the “previously at risk” or “not previously at risk” subpopulation to which the individual belongs.

In practice, this proposal means that we stratify by the interaction between risk prior to the start of follow-up and the number of prior episodes. Therefore, the use of the individual random error term *υi* intends to capture the error that will be made when imputing, as well as the effect of any variable with a non-nil effect not being considered in the analysis. Stratifying by the number of prior episodes intends to safeguard event dependence: doing it as an interaction with the fact that it is an individual previously at risk, or not, separates the two subpopulations to avoid mixing times that are not comparable on the same scale.

To impute the prior history, a generalized linear model (GLM) based on the Conway–Maxwell Poisson distribution (COMPoisson) [20] is fitted to the observed number of events. Imputed values are randomly sampled from the corresponding distribution with the parameters obtained in the previous step, including random noise generated from a normal distribution. To produce proper estimation of uncertainty, the described methodology is used within a multiple imputation framework. The data in the *Results* section reflect the combination of 5 imputed data sets (following the classic advice to use a low number of imputed data sets and considering that there is little to gain from using more [21–23]), according to Rubin’s rules [23] and based on the following steps in a Bayesian context:

1. Fit the corresponding COMPoisson model and find the posterior mean $\hat{\beta}$ and variance $V\left(\hat{\beta}\right)$ of model parameters *β*.
2. Draw new parameters *β*^∗^ from $N\left(\hat{\beta,}V\left(\hat{\beta}\right)\right)$.
3. Draw imputations from the COMPoisson distribution using the scores obtained in the previous step as parameters.

A detailed description of the whole imputation process can is reported by Hernández et al. (2020) [24].

### Simulation study

Six populations were simulated through the R package, survsim [25], corresponding to previously described cohorts of workers [19, 26]. The first three simulated worker history depending on the occurrence of long-term sick leave with several intensities of event dependence. Given that the simulations are carried out through Weibull distributions with ancillary parameters equal to 1 (that is, exponential distributions) the hazard was deemed constant within each episode, but different between episodes (in other words, there was event dependence). For the fourth, fifth, and sixth populations, the outcome was sick leave according to diagnosis (i.e., respiratory system, musculoskeletal system, and mental and behavioral disorders). In this case, the hazard functions were not constant within the episode.

Table 1 shows the characteristics of each episode in each population. The maximum number of episodes that a subject may suffer was not fixed, but the baseline hazard was considered constant when *k ≥ 3*. The exposure is represented by covariates *X*_*1*_*, X*_*2*_*,* and *X*_*3*_, with *X*_*j*_ *≈ Bernoulli (0.5) and β*_*1*_ *= 0.25, β*_*2*_ *= 0.5,* and *β*_*3*_ *= 0.75* representing effects of different magnitudes set independently of the episode *k* to which the worker is exposed.

**Table 1:** Characteristics of the simulated populations

Episode	Distribution	***β***_***0***_	Ancillary	HR
1	Weibull	8.109	1	1
2	Weibull	7.927	1	1.20
≥ 3	Weibull	7.745	1	1.44
1	Weibull	8.109	1	1
2	Weibull	7.703	1	1.50
≥ 3	Weibull	7.298	1	2.25
1	Weibull	8.109	1	1
2	Weibull	7.193	1	2.50
≥ 3	Weibull	6.276	1	6.25
1	Log-normal	7.195	1.498	1
2	Log-logistic	6.583	0.924	1.77
≥ 3	Weibull	6.678	0.923	2.53
1	Log-logistic	7.974	0.836	1
2	Weibull	7.109	0.758	3.81
≥ 3	Log-normal	5.853	1.989	7.19
1	Log-normal	8.924	1.545	1
2	Log-normal	6.650	2.399	10.13
≥ 3	Log-normal	6.696	2.246	11.19

Weibull distribution: $$f(t)={\lambda pt}^{p-1}{e}^{-\lambda {t}^p},\lambda ={e}^{-p{\beta}_0}$$

Lognormal distribution: $$f(t)=\frac{1}{t\sigma \sqrt{2\pi }}{e}^{\left[\frac{-1}{2{\sigma}^2}{\left\{\log (t)-\mu \right\}}^2\right]},\mu ={\beta}_0$$

Loglogistic distribution: $$f(t)=\frac{\lambda^{1/\gamma }{t}^{1/\gamma -1}}{\gamma {\left\{1+{\left(\lambda t\right)}^{1/\gamma}\right\}}^2},\lambda ={e}^{-{\beta}_0}$$

Four different situations, which were combinations between two possible follow-up times (2 and 5 years) and two maximum times at risk prior to the beginning of the cohort (2 and 10 years), were simulated for each population. For example, for 10 years’ maximum time at risk and 5 years’ follow-up, we generated dynamic populations with 15 years’ history and selected subjects who were either present at 10 years’ follow-up or incorporated after that date. Follow-up time was then re-scaled, setting *t = 0* at 10 years for subjects already present in the population, and setting *t = 0* as the beginning of the follow-up period for those incorporated later. This procedure allowed us to obtain a cohort in which some subjects had worked up to 10 years and were treated as unknown with 5 years of effective follow-up (observed between 10 and 15 years in the original simulated follow-up). In each case, *m = 100* samples of sizes 250, 500, and 1000 were simulated with different proportions of subjects at risk prior to entering the cohort (0.1, 0.3, 0.5, 0.75 and 1).

Our proposal was compared to a model with frailty and common baseline hazard in terms of the number of previous events, but with different risk per subpopulation. We named this the “Common Hazard Frailty Model with stratification by subpopulation” (COMMON). Importantly, this model does not take event dependence into account, as expressed in Eq. 6, but separates individuals by prior risk:

9 $${\lambda}_{ir}(t)={\nu}_i{\lambda}_{0r}(t){e}^{X_i\beta }$$

Additionally, a second simulation study was conducted with the same technical settings but with *m* = 1000 simulation instances to avoid spurious fluctuations and with only one covariate with no effect *(β = 0)*, to assess type I error rates for rejecting the null hypothesis of no exposure effect. Table 2 presents the performance measures used for evaluating the different methods.

**Table 2:** Performance evaluation criteria

Simulation study 1	Evaluation criteria ***(covariate j = 1, 2, 3 and sample m = 1, …, 100)***
Mean relative bias	$\frac{\sum_{j=1}^3\sum_{m=1}^{100}\frac{\left|{\hat{\beta}}_{jm}-{\beta}_j\right|}{\beta_j}\times 100}{3\times 100}$
Average length of 95%CI	$\frac{\sum_{j=1}^3\sum_{m=1}^{100}2{Z}_{1-\alpha /2} SE\left({\hat{\beta}}_{jm}\right)}{3\times 100}$
Coverage	Percentage of times the 95% confidence interval ${\hat{\beta}}_{jm}\pm {Z}_{1-\alpha /2} SE\left({\hat{\beta}}_{jm}\right)$ includes β_*j*_
**Simulation study 2**	**Evaluation criterium**
Type I error	Percentage of times the *H*_*0*_*:β = 0* is rejected, for *m =* 1, …, 1000

## Results

**Fig. 2:**
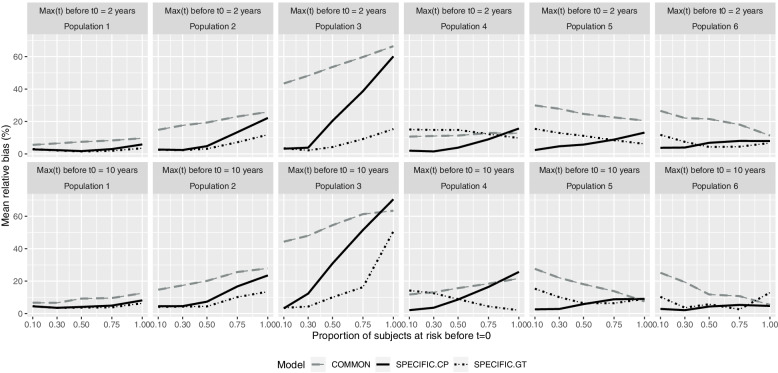
Bias according to population and maximum time at risk prior to the beginning of the cohort

The presented results refer to *n* = 1000 and 5 years’ follow-up because the differences observed for *n* = 250, *n* = 500, and 2 years’ follow-up were not considered relevant (see Supplementary Material). Regarding bias, Fig. 2 highlights that the COMMON model only obtained values under 10% in population 1 and in some cases when 100% of individuals were at prior risk. Models SPECIFIC.CP and SPECIFIC.GT generally obtain biases < 10% in most situations, except for SPECIFIC.GT in populations 4 and 5, which is slightly above this threshold, and for SPECIFIC.CP, especially in population 3 when 100% of individuals had prior risk at *t = 0*, which seemed more sensitive. For the first three populations, SPECIFIC.GT shows equal or less bias than SPECIFIC.CP, whereas the converse is true for the second three populations, provided that risk starts during follow-up for at least 50% of the cohort.

**Fig. 3:**
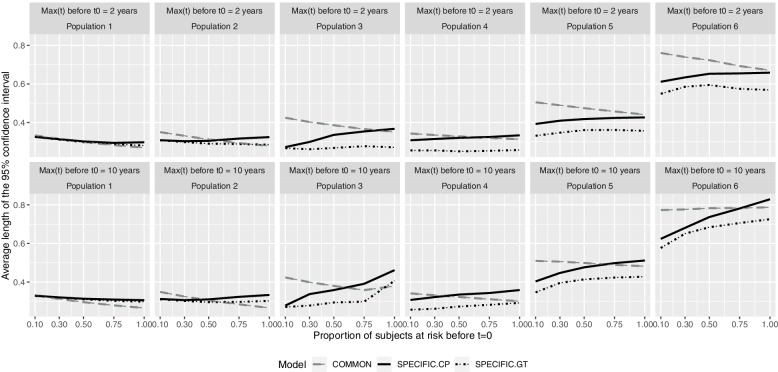
Average length of the 95% confidence interval according to population and maximum time at risk prior to the beginning of the cohort

The average 95%CIs are largest in the COMMON model, excluding population 1, when up to 30% of individuals were at previous risk, always overcoming the accuracy of the SPECIFIC.CP model in populations 1, 2, and 3, with the SPECIFIC.GT model most accurate in populations 4, 5, and 6 (Fig. 3).

**Fig. 4:**
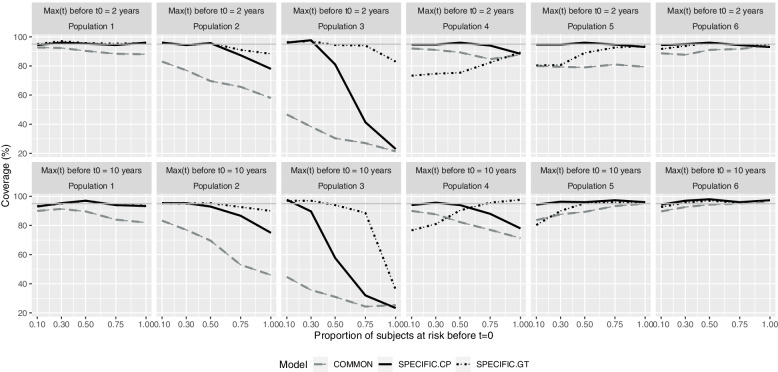
Coverage according to population and maximum time at risk prior to the beginning of the cohort

The 95%CI coverage of the COMMON model was clearly under 95% in most cases, decreasing as the event dependence increases, and for the first four populations, the proportion at risk prior to *t = 0*. The coverages for models SPECIFIC.CP and SPECIFIC.GT were generally acceptable, even when excessively conservative in some cases (> 95%). The SPECIFIC.CP model failed in population 3 when 100% had prior risk, due to the high bias, whereas the SPECIFIC.GT model obtained coverage < 80% even in population 4 when < 100% of individuals were at prior risk (Fig. 4).

Tables S1 and S2 (Supplementary Material) summarize the results using arbitrary relative bias (< 10%) and coverage (92.5–97.5%) criteria. Considering all situations, the COMMON model rarely met these criteria, while the SPECIFIC.CP model showed the highest level of compliance.

**Fig. 5:**
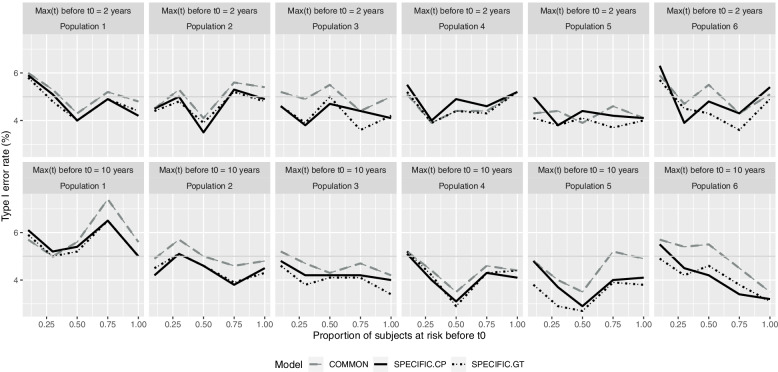
Type I error rate according to population and maximum time at risk prior to the beginning of the cohort

Figure 5 shows the type I error distribution for each method, with acceptable rates of around 5% observed in all cases.

## Discussion

Our results indicate that not knowing the number of prior episodes an individual has experienced should not justify the use of models with a common baseline hazard, which in most situations show higher bias and less coverage than specific baseline hazard models. The first step, which is indispensable for choosing the correct method, should be to describe the baseline hazard form of the phenomenon under study in each episode. The SPECIFIC.GT model should be selected if this is a function of constant risk, while the SPECIFIC.CP model would also be acceptable when information about the number of prior episodes is lacking in up to 30–50%; however, the SPECIFIC.CP model should be selected for phenomenon ruled non-constant when there is a lack of prior information in up to 50% of cases. Neither model is sufficiently robust if the number of previously experienced episodes is unknown for all individuals, even though the SPECIFIC.GT model could be adequate in some cases.

Some readers may be surprised by the finding that bias for the COMMON models in populations 5 and 6 decreased when increasing the proportion of workers at risk before *t = 0*. This may be because these populations present high levels of recurrence: thus, more subjects are at risk prior to *t = 0* meaning that more subjects will have already had ≥2 episodes (and will be imputed with values ≥2). Given that our simulation set had the same baseline hazard from the second episode onwards (a realistic convergence phenomenon), many subjects will have had the same baseline hazard and the COMMON model will have performed better.

Other unexpected results are seen with the partially non-monotonous bias and CI coverage functions associated with increasing the proportion of missing data (e.g., the SPECIFIC.CP and SPECIFIC.GT models in population 1). These situations were in the minority, and the reader should keep in mind that the actual differences were very low despite appearing relevant (the Y-axis scales in some figures are very accurate). The results for *n* = 250 and *n* = 500 (Supplementary Material) did not follow the same pattern, which indicates that these differences are probably spurious.

Finally, it is important to note that the effectiveness of imputing prior missing episodes to account for left-censored data depends strongly on the assumption of ‘missing at random’ (i.e., that measured covariates actually predict the episode history of an individual). Therefore, a correctly specified imputation (and similarly, an appropriate final survival model specification) is essential to obtain unbiased and efficient estimates.

## Conclusions

This simulation study reveals that simply ignoring prior episodes of the outcome of interest and using common baseline hazard models (the most common approach in epidemiological cohort studies) can lead to extremely biased and inefficient estimates. In turn, this will generate inaccurate conclusions, especially when the amount of missing information is high. However, the performance of these estimates can be improved by using the proposed methodology, which is already available on standard software and ready to be used by any interested researcher as R package *miRecSurv* [27].

## Supplementary Information

**Additional file 1.**

**Additional file 2.**

## Data Availability

R codes used in the described analyses are available as supplementary material and in the GitHub repository https://github.com/dmorinya/miRecSurv.

## Abbreviations

COMMON: Common hazard frailty model with stratification by subpopulation
CI: Confidence interval
COMPoisson: Conway-Maxwell Poisson
CP: Counting process
GLM: Generalized linear model
GT: Gap time
HR: Hazard ratio
PWP: Prentice, Williams, and Peterson model
SPECIFIC.CP: Specific hazard frailty model imputed (Counting process formulation)
SPECIFIC.GT: Specific hazard frailty model imputed (Gap Time formulation)
