# Supplementary material for: Left-censored recurrent event analysis in epidemiological studies: a proposal for when the number of previous episodes is unknown
Source: BMC Med Res Methodol. 2022 Jan 16;22:20. doi: 10.1186/s12874-022-01503-1 (PMC8761288; doi:10.1186/s12874-022-01503-1)

Supplementary material

Figure 1s. Relative bias (n=500 and follow-up=5 years)

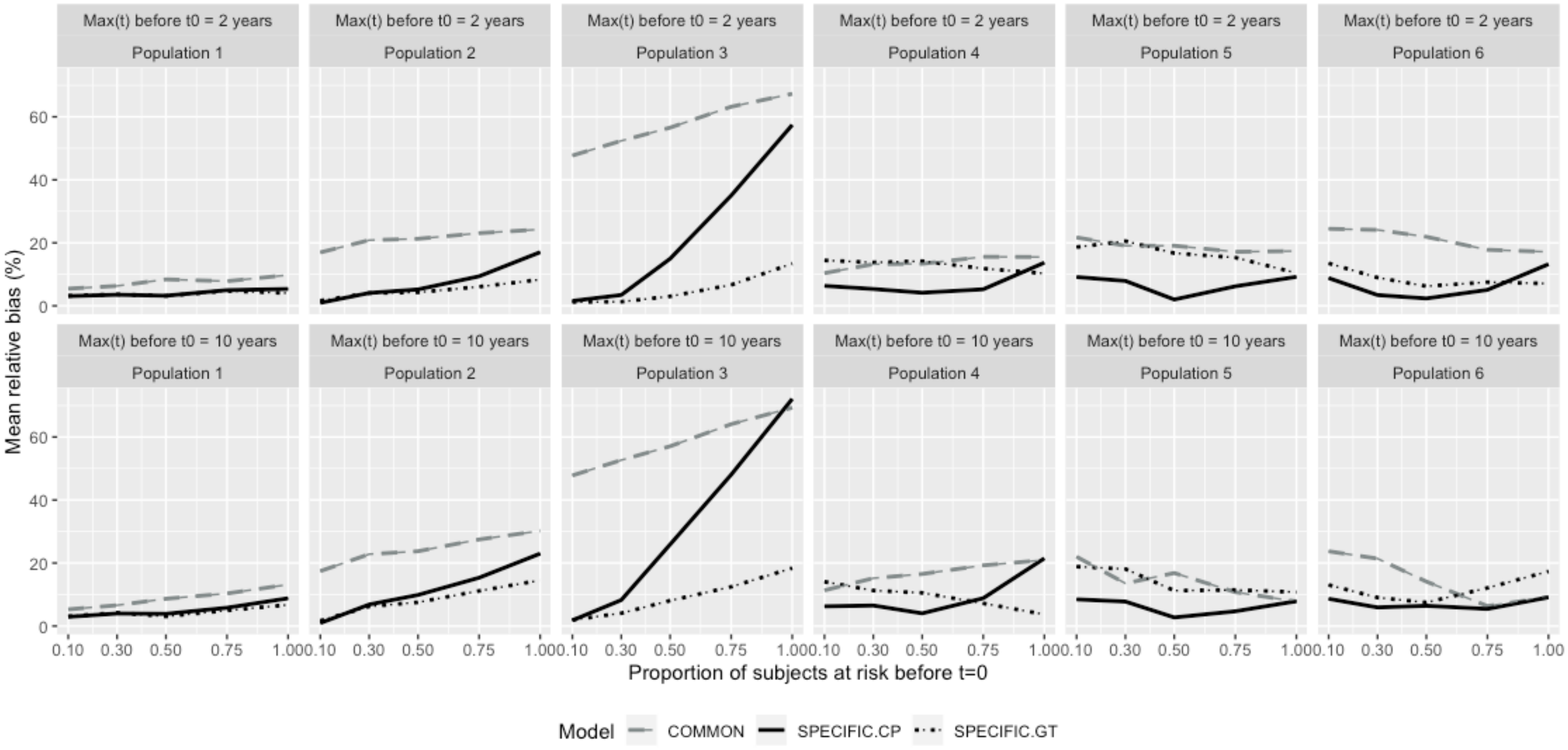

Figure 2s. Average length of the 95% confidence interval (n=500 and follow-up=5 years)

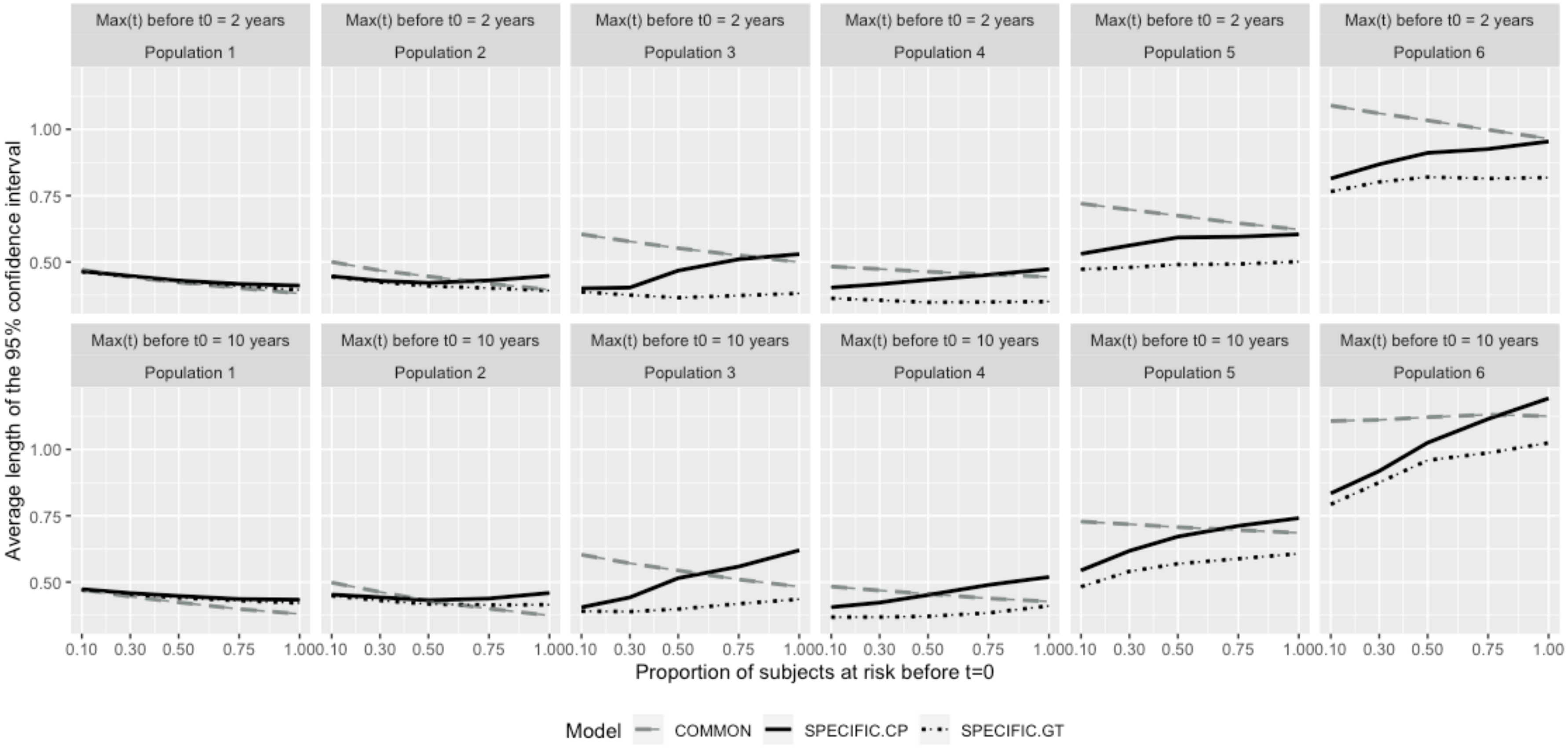

Figure 3s. Coverage of the 95% confidence intervals (n=500 and follow-up=5 years)

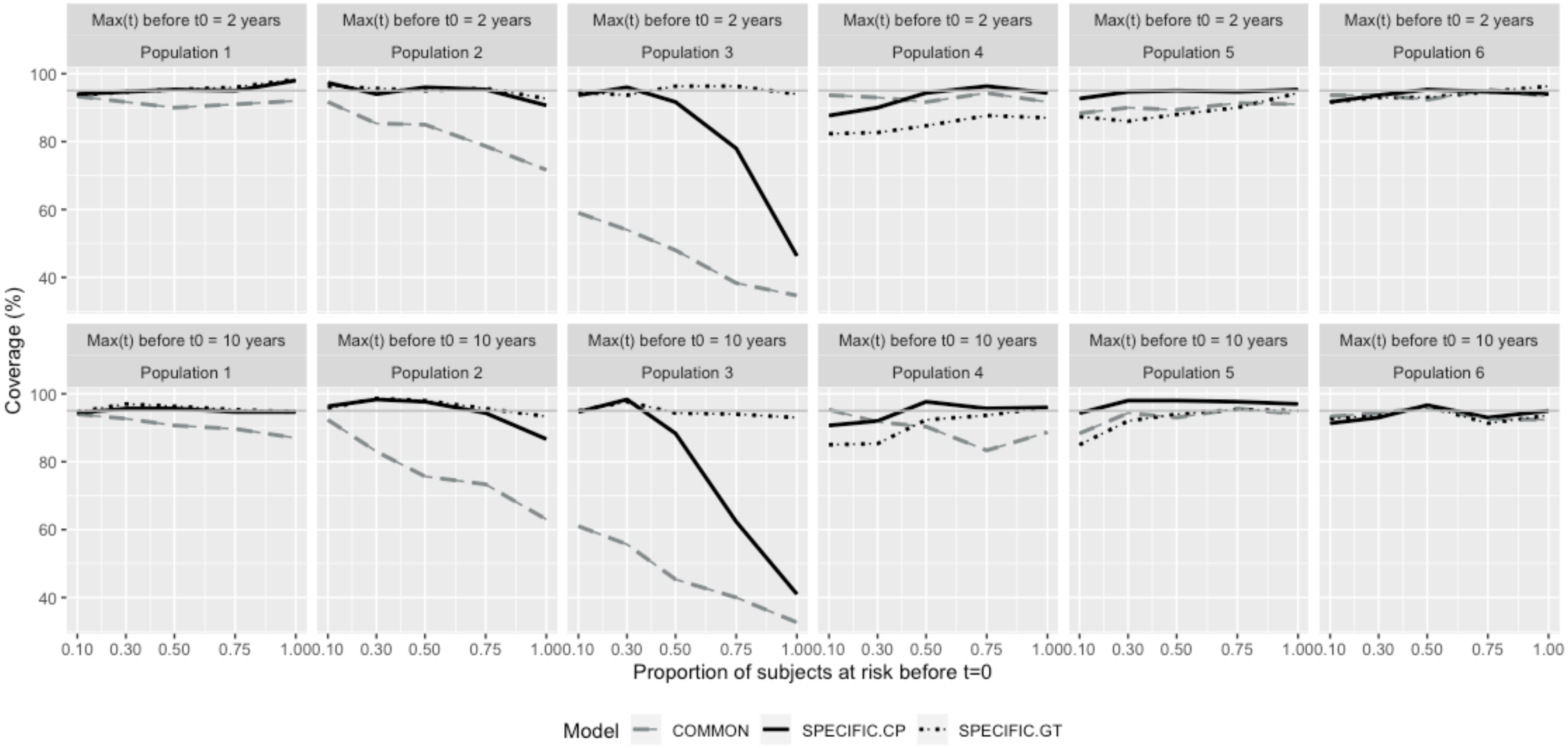

Figure 4s. Error type I rate (n=500 and follow-up=5 years)

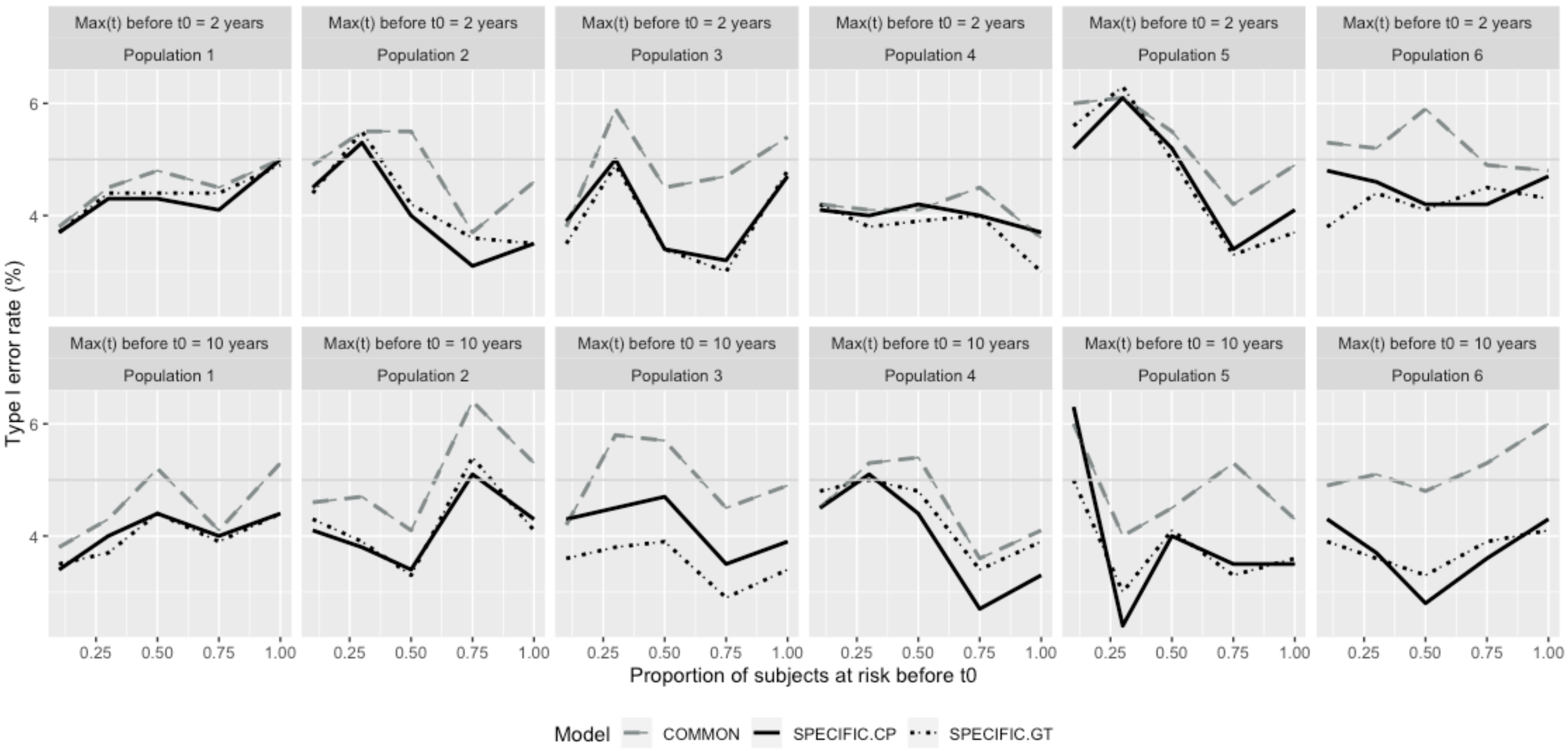

Figure 5s. Relative bias (n=250 and follow-up=5 years)

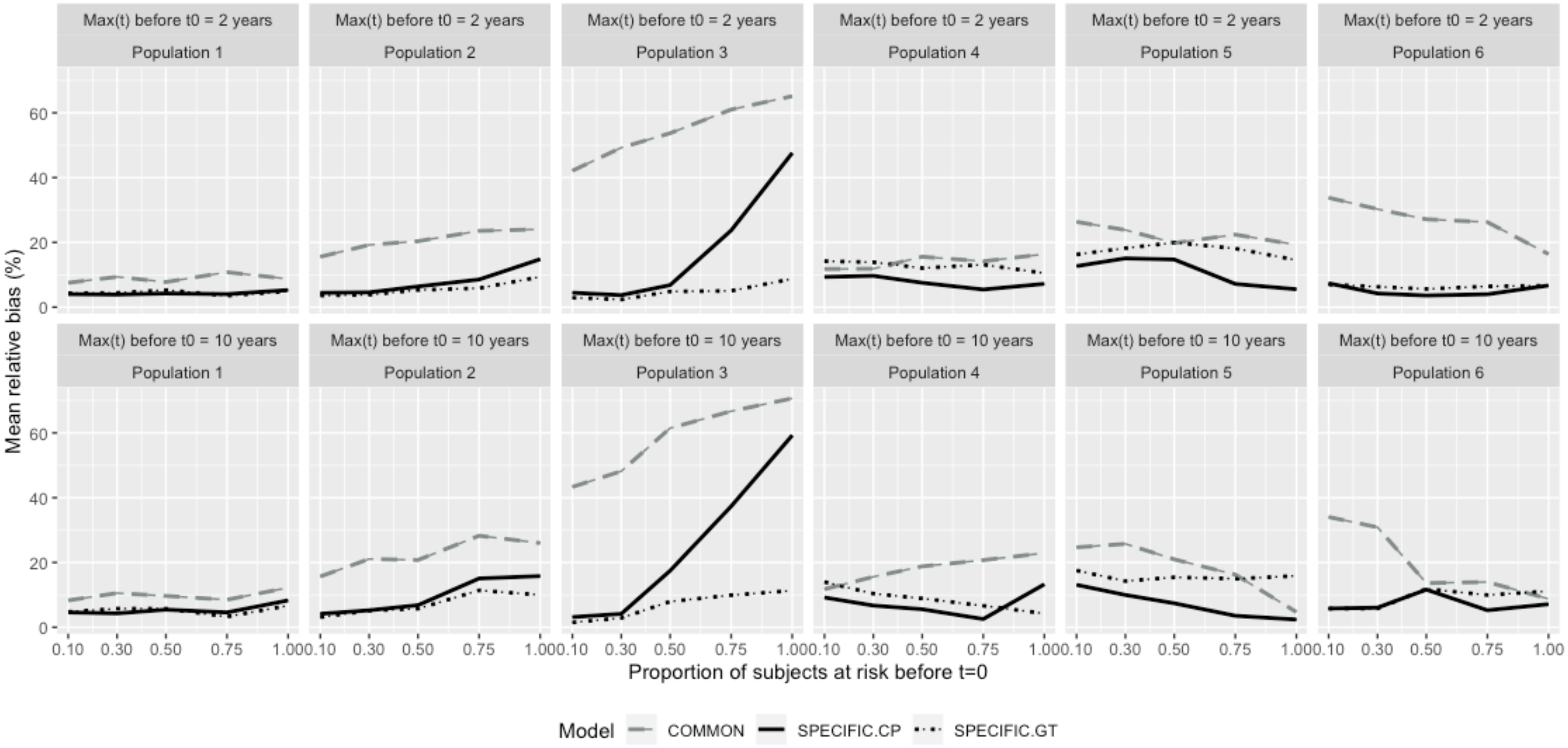

Figure 6s. Average length of the 95% confidence interval (n=250 and follow-up=5 years)

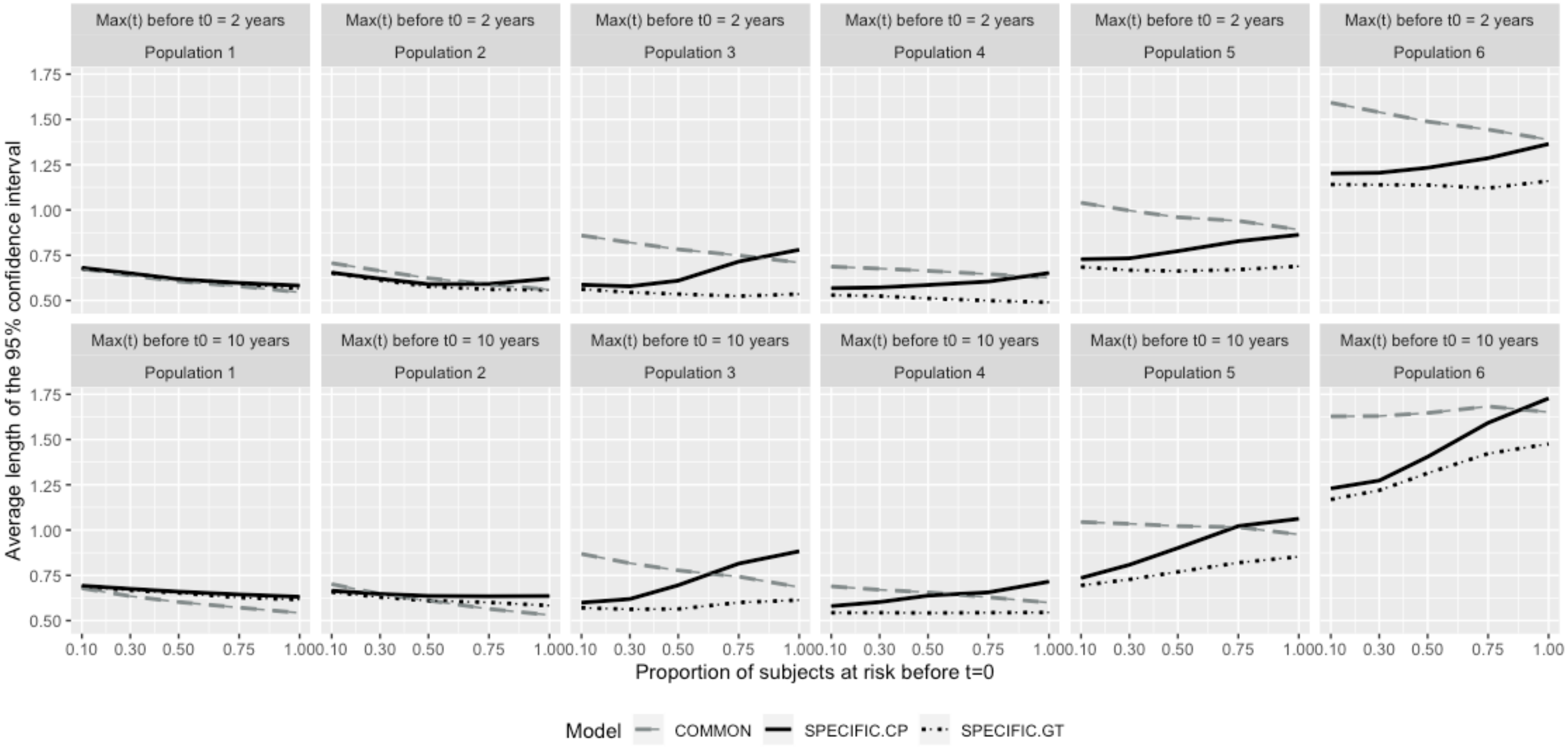

Figure 7s. Coverage of the 95% confidence intervals (n=250 and follow-up=5 years)

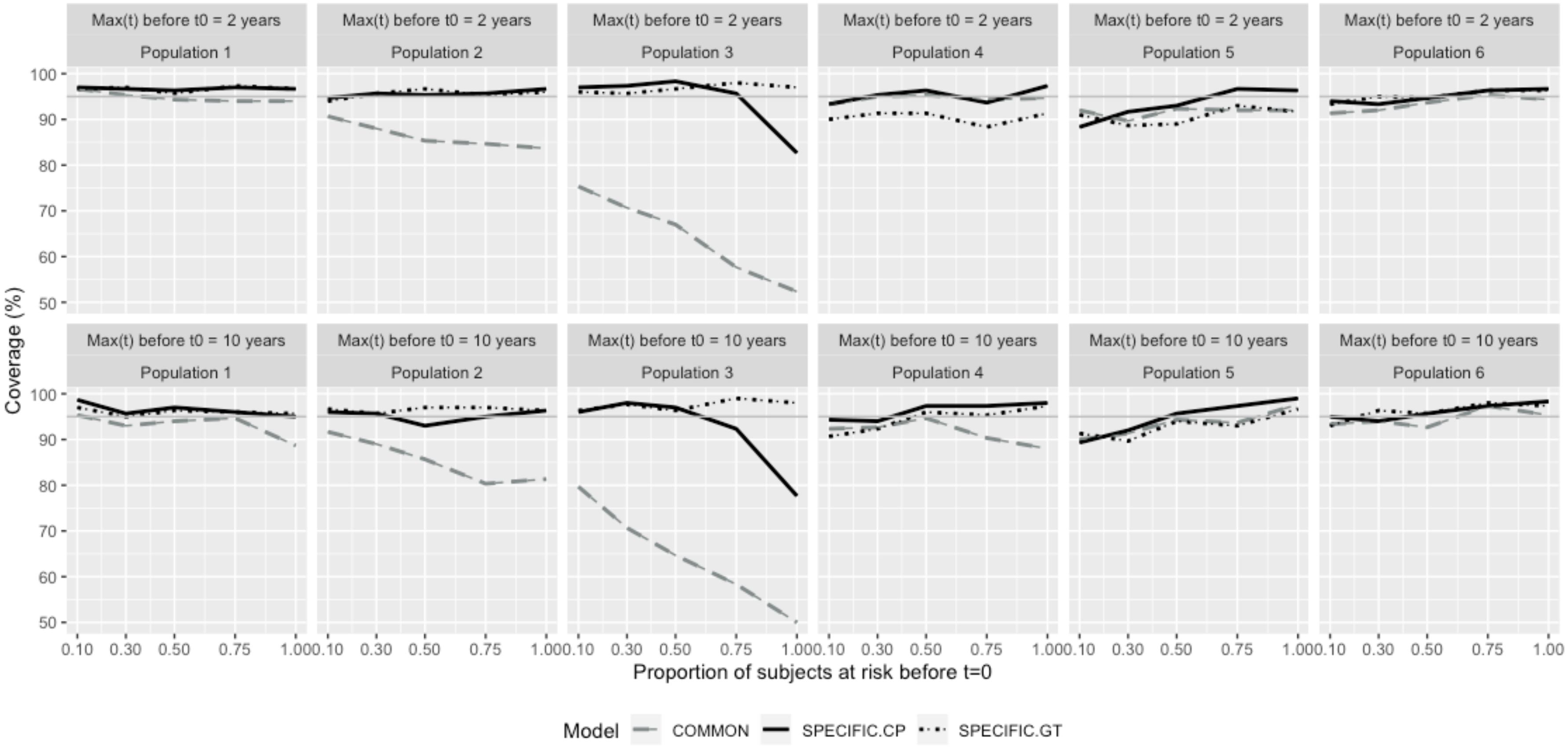

Figure 8s. Error type I rate (n=250 and follow-up=5 years)

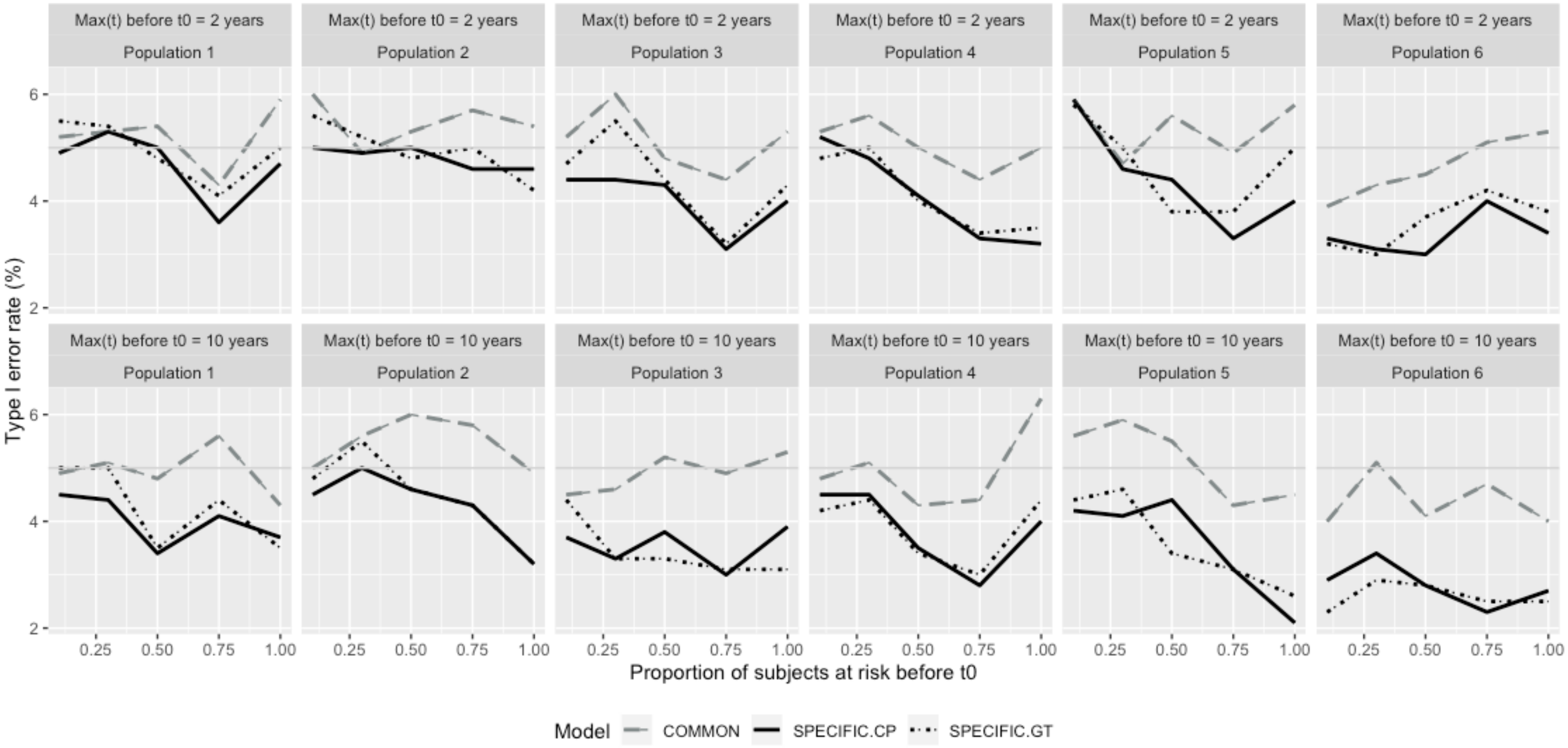

Figure 9s. Relative bias (n=1000 and follow-up=2 years)

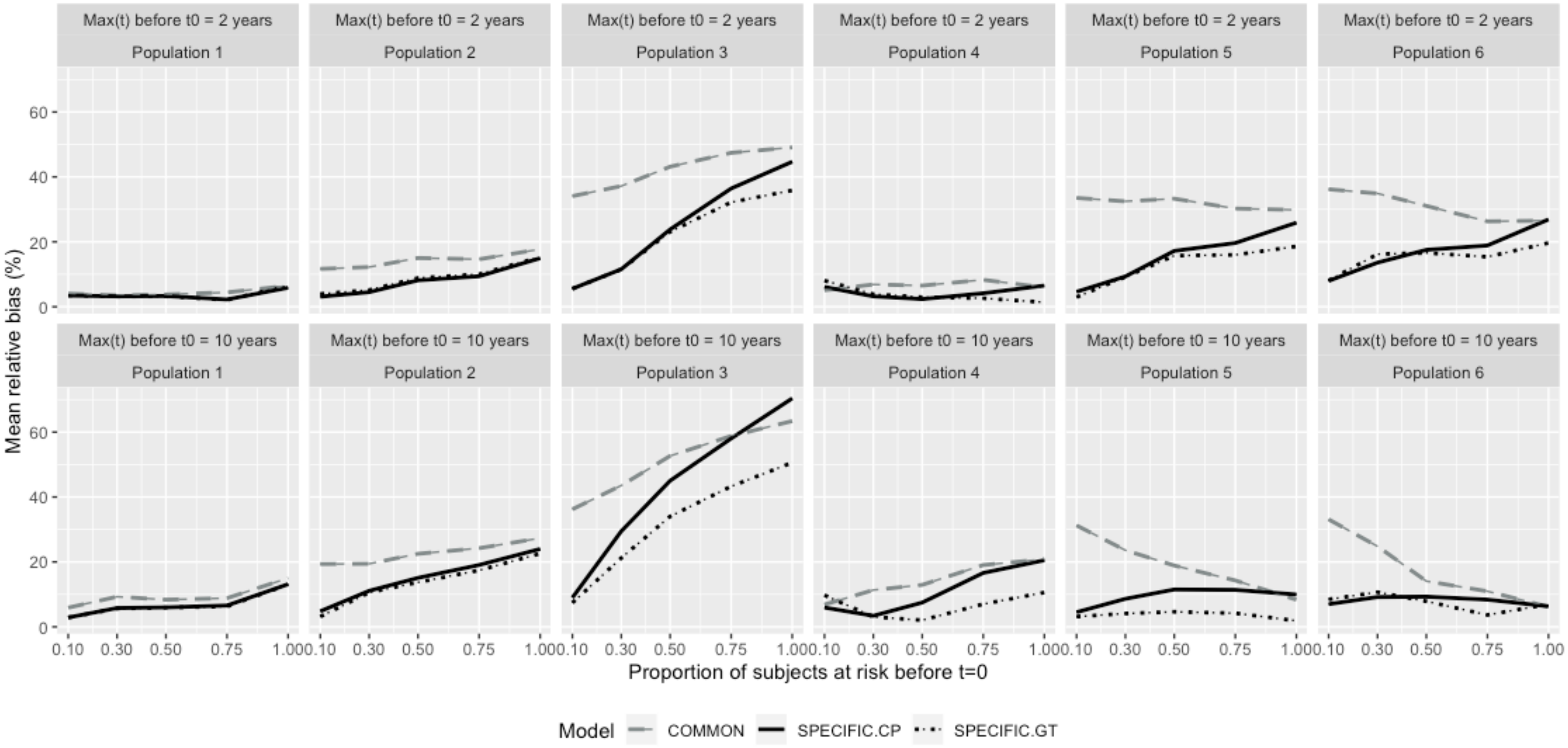

Figure 10s. Average length of the 95% confidence interval (n=1000 and follow-up=2 years)

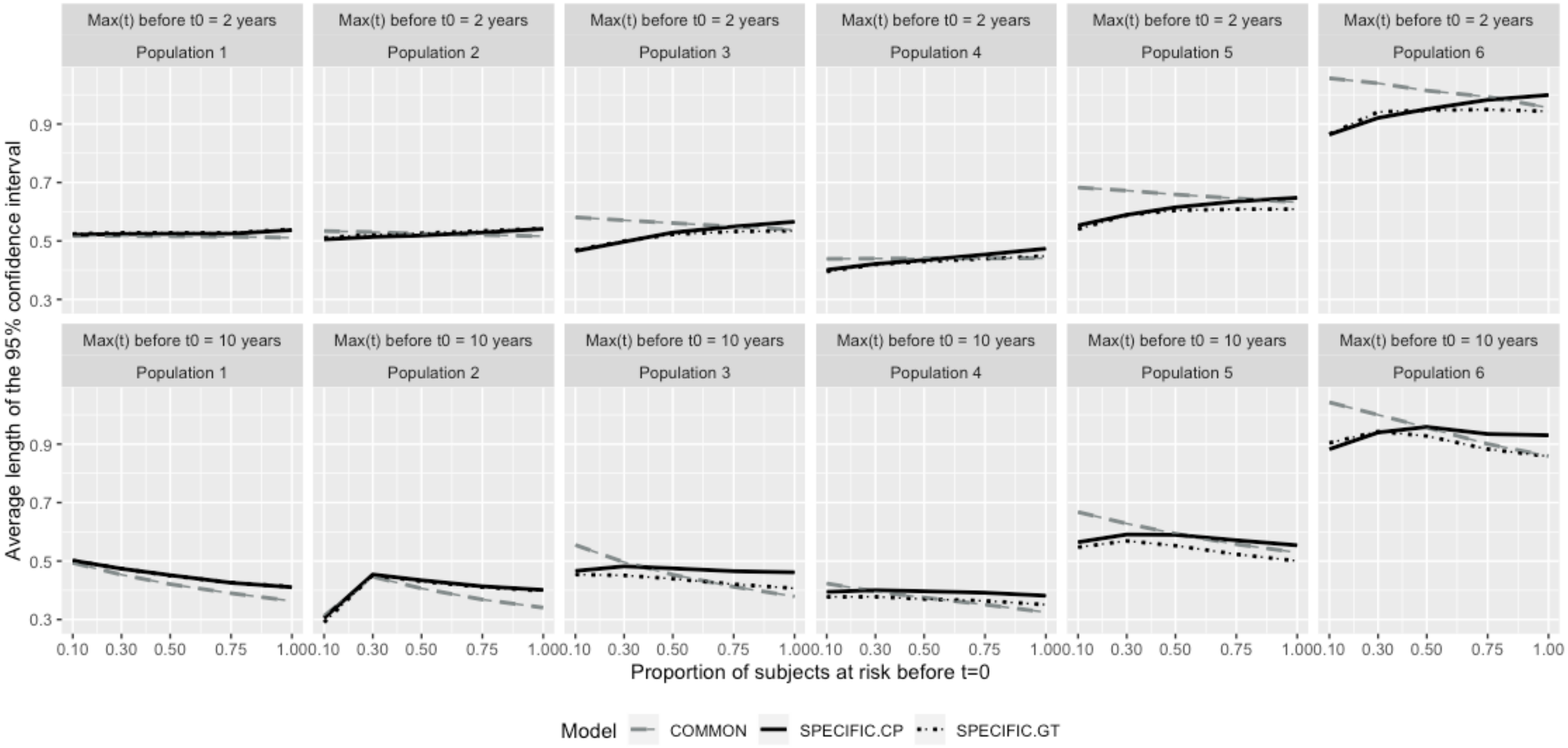

Figure 11s. Coverage of the 95% confidence intervals (n=1000 and follow-up=2 years)

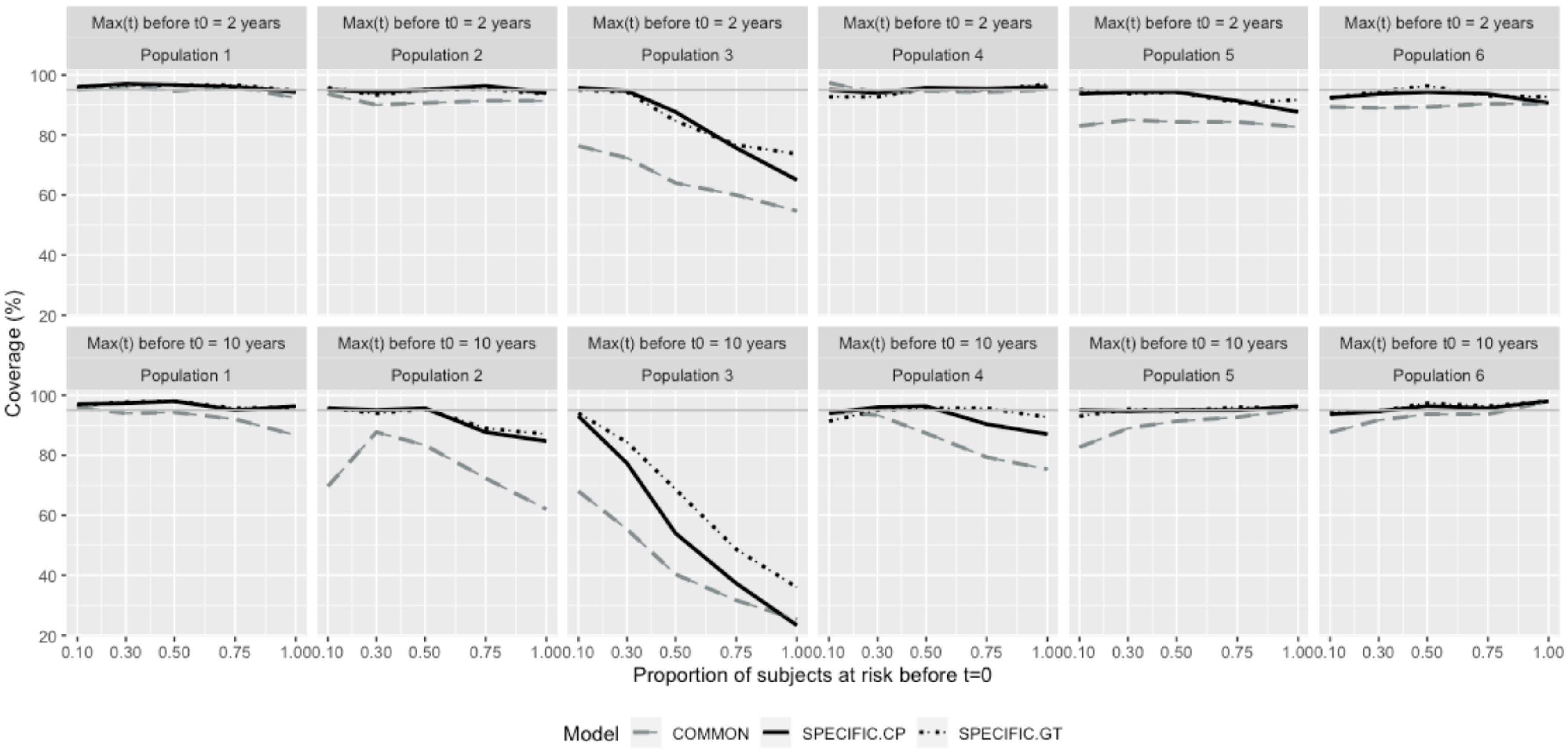

Figure 12s. Error type I rate (n=1000 and follow-up=2 years)

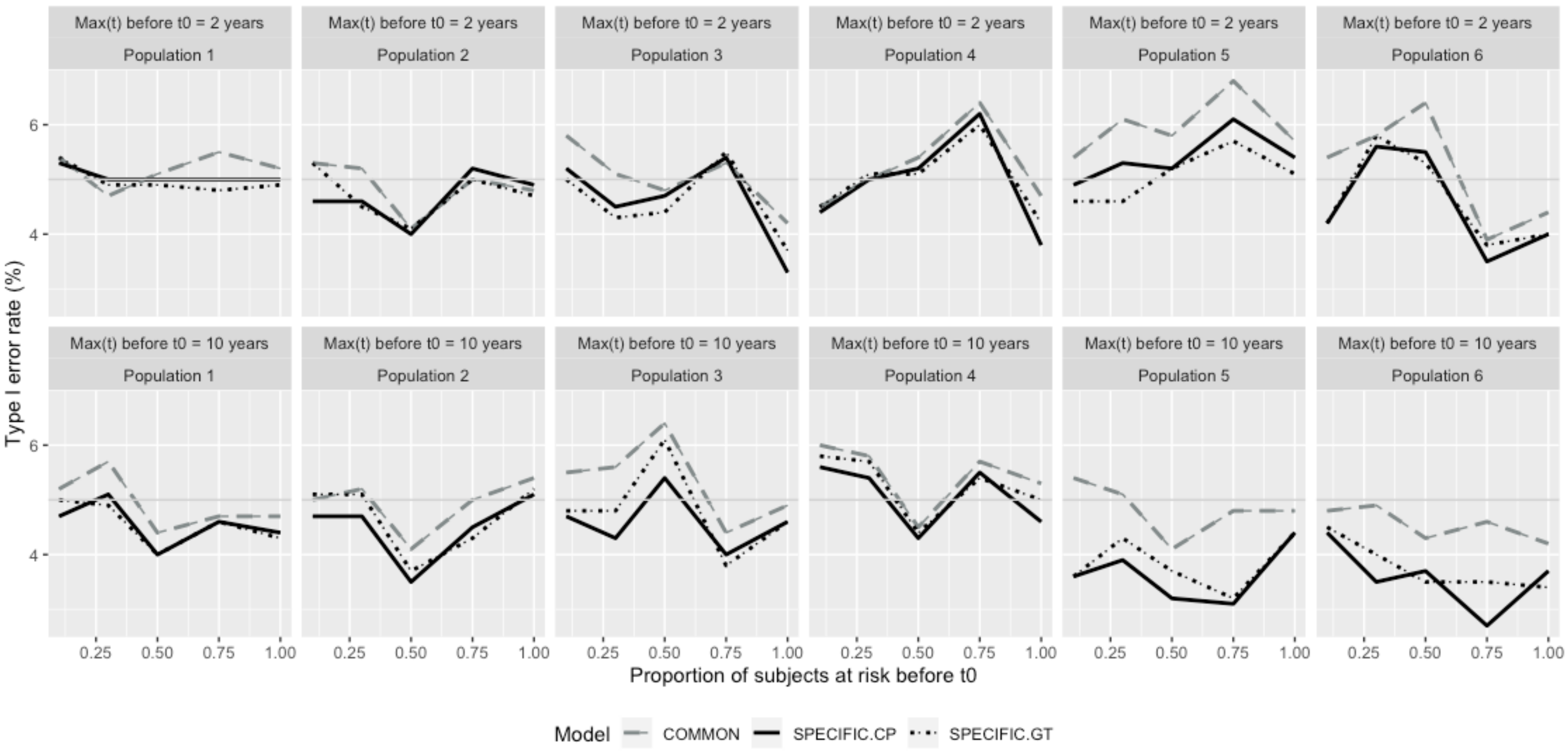

Figure 13s. Relative bias (n=500 and follow-up=2 years)

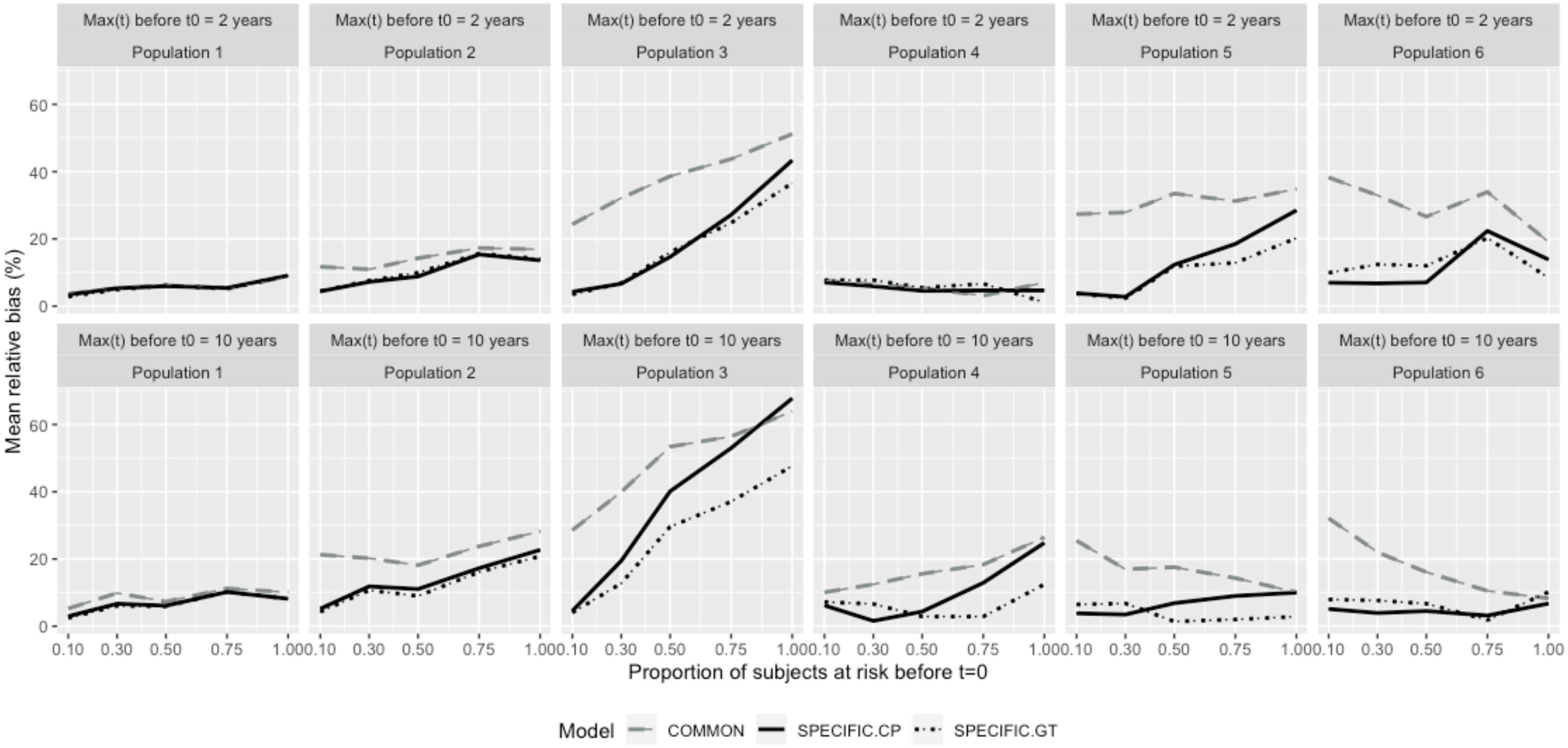

Figure 14s. Average length of the 95% confidence interval (n=500 and follow-up=2 years)

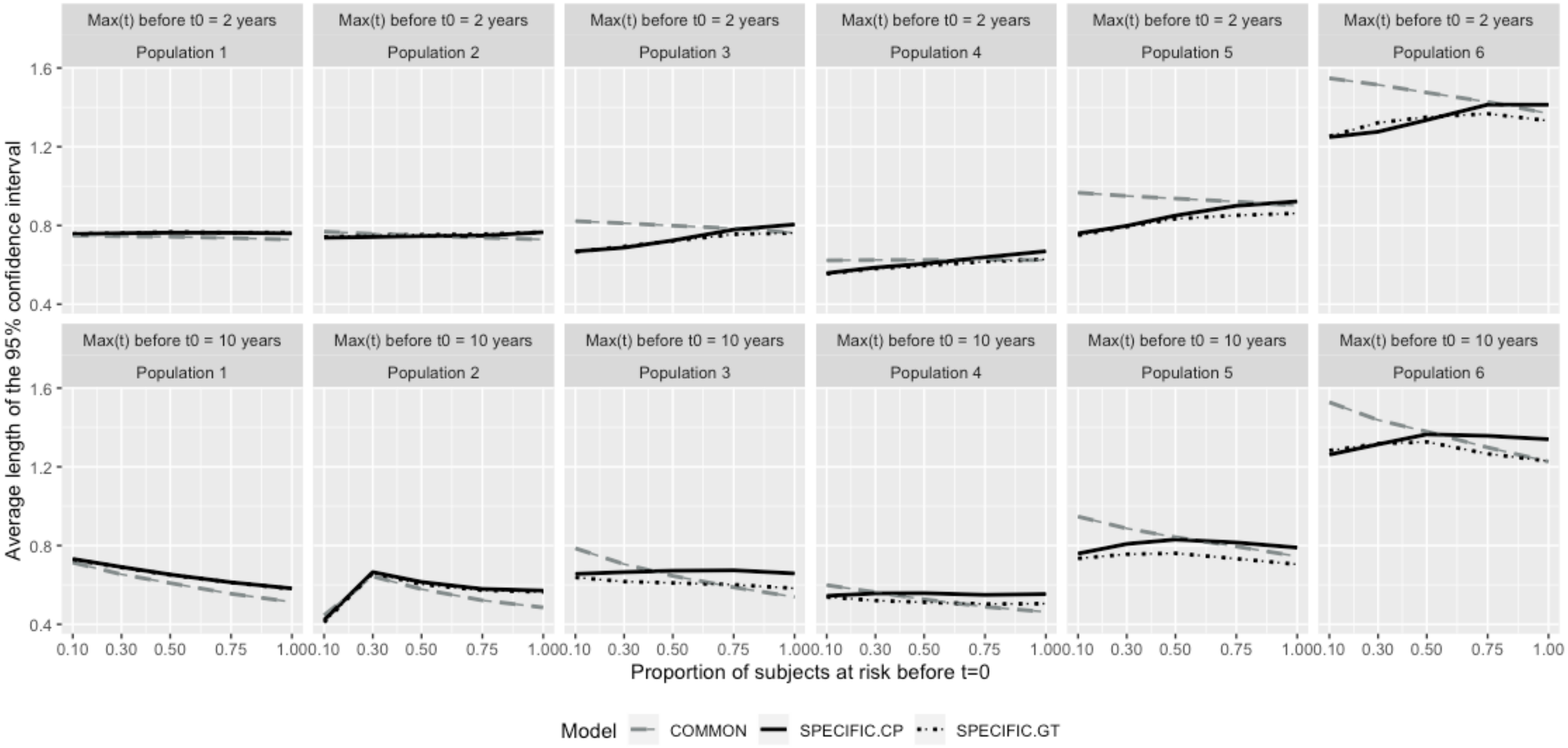

Figure 15s. Coverage of the 95% confidence intervals (n=500 and follow-up=2 years)

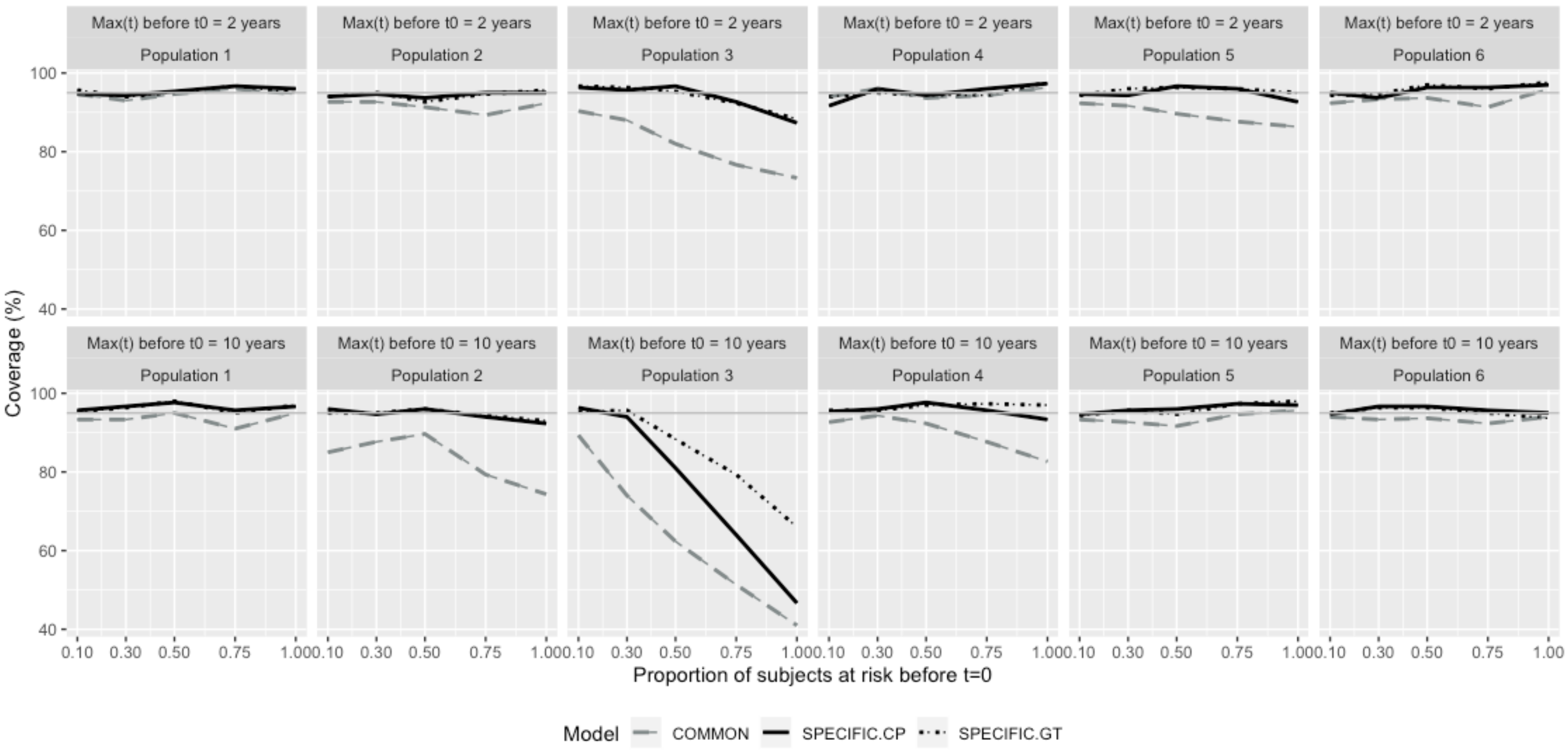

Figure 16s. Error type I rate (n=500 and follow-up=2 years)

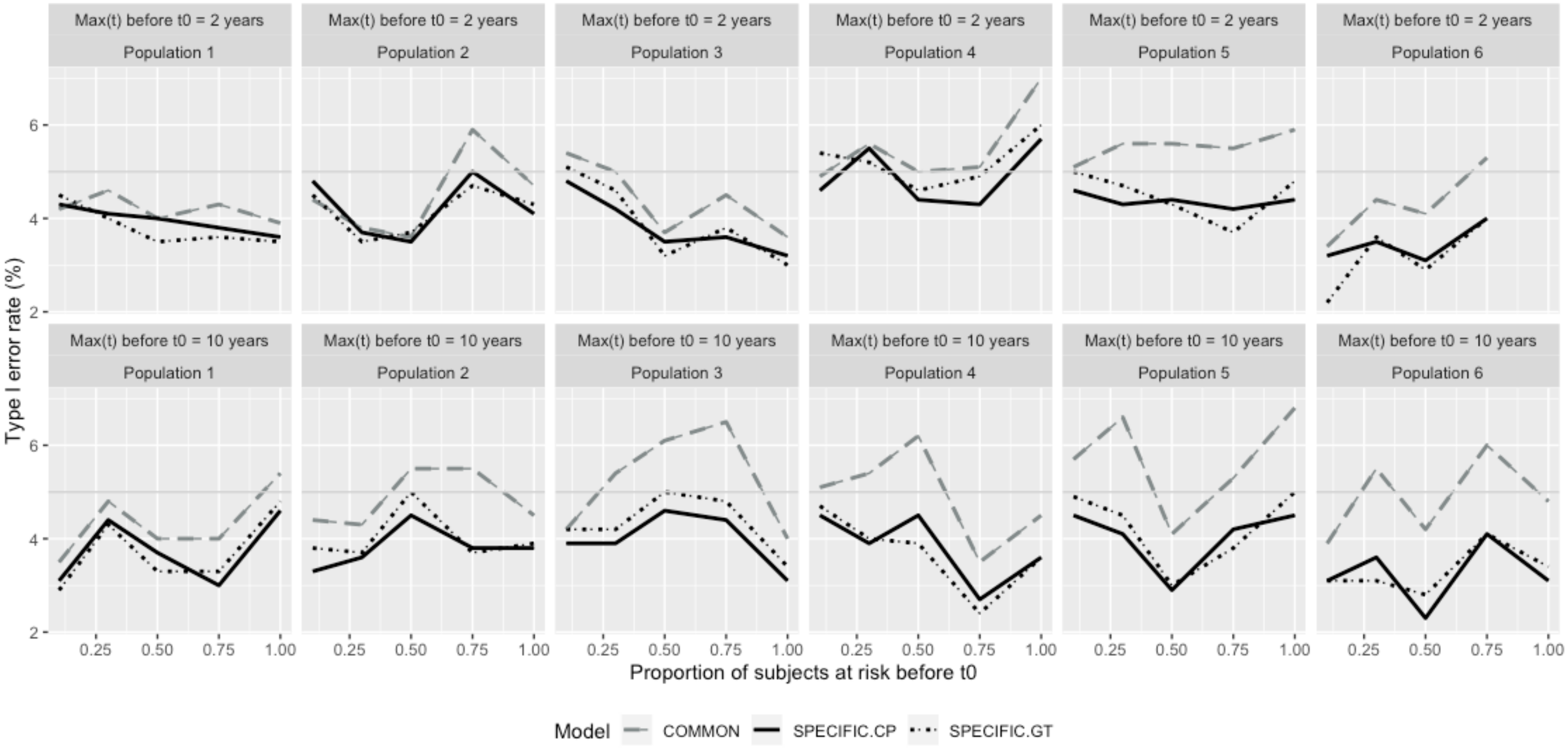

Figure 17s. Relative bias (n=250 and follow-up=2 years)

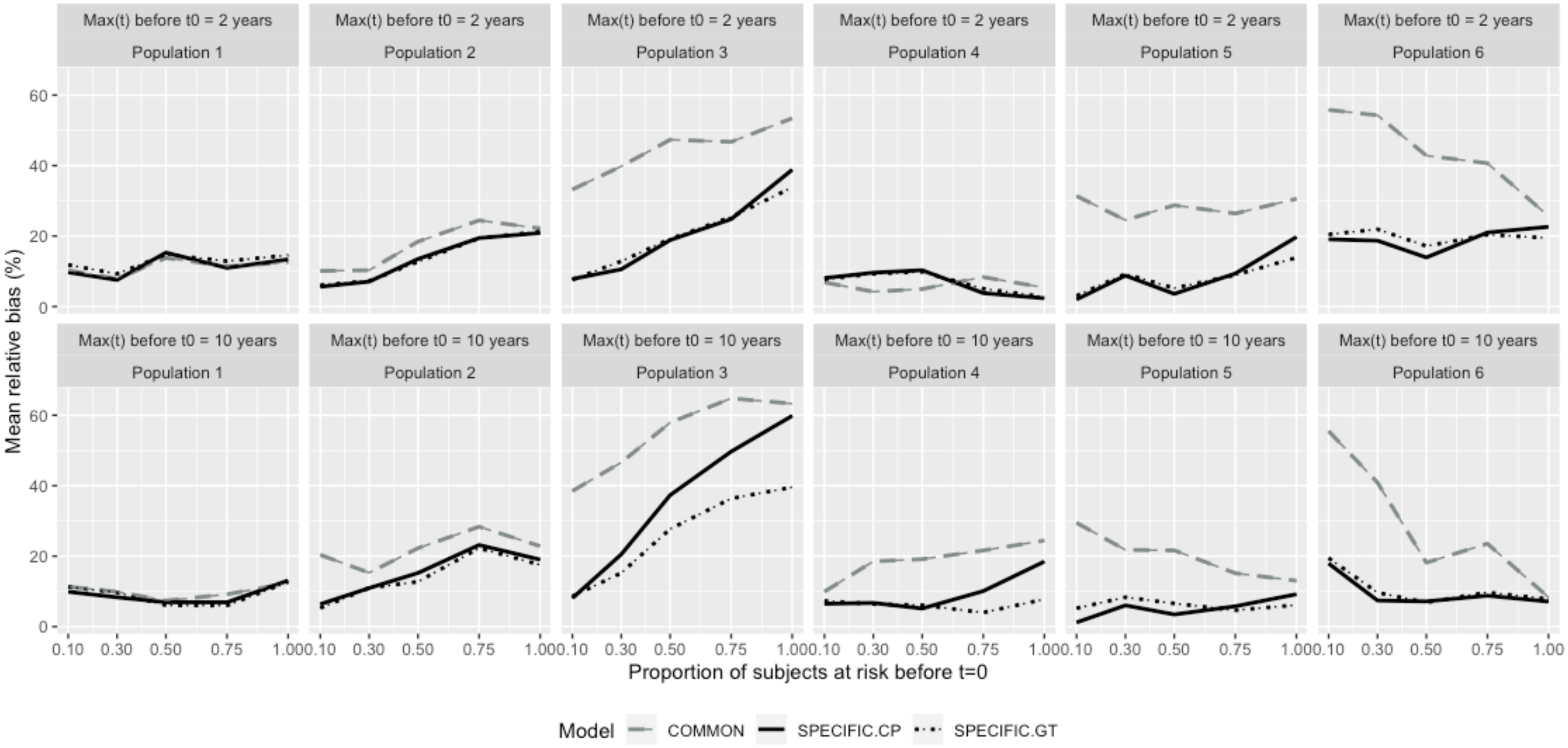

Figure 18s. Average length of the 95% confidence interval (n=250 and follow-up=2 years)

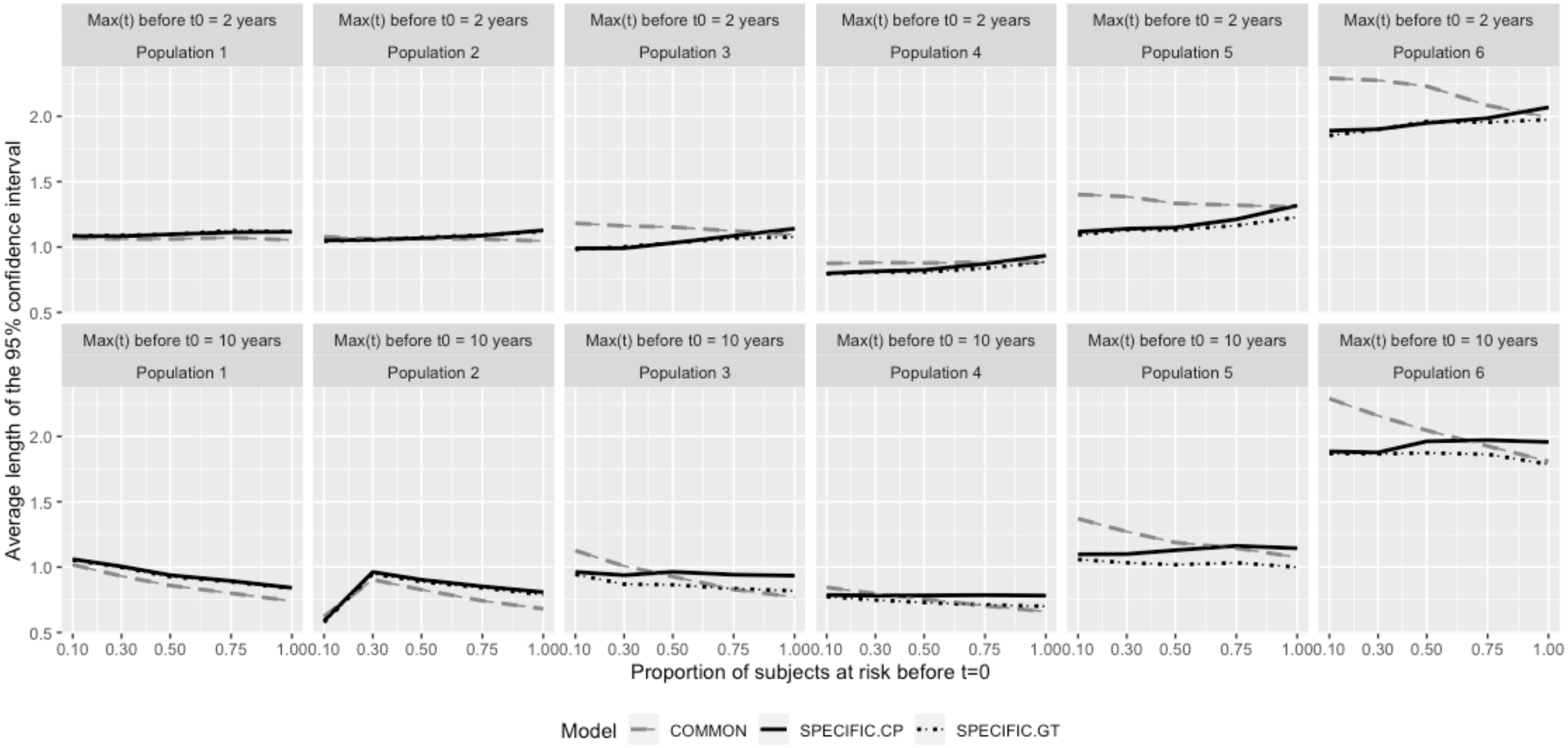

Figure 19s. Coverage of the 95% confidence intervals (n=250 and follow-up=2 years)

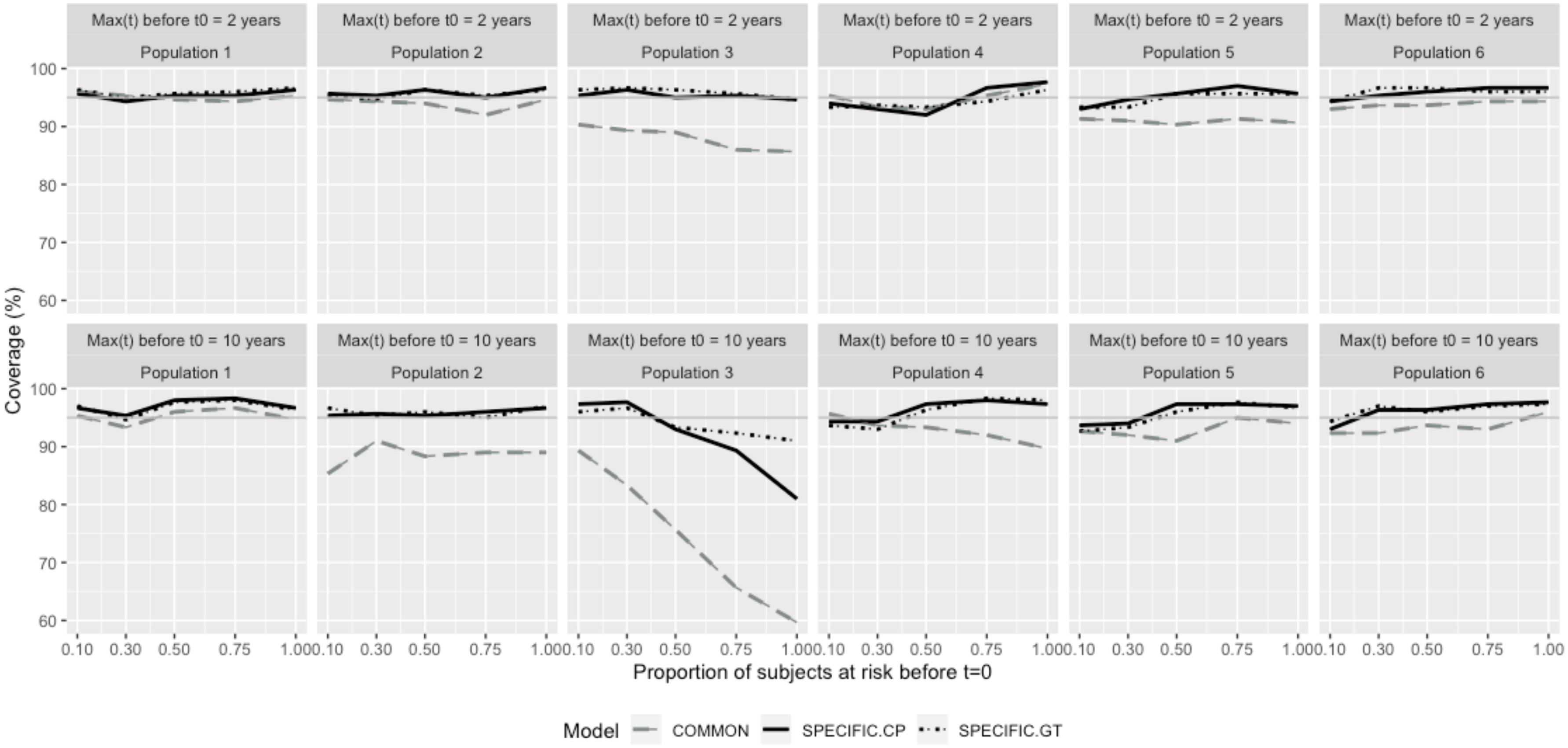

Figure 20s. Error type I rate (n=250 and follow-up=2 years)

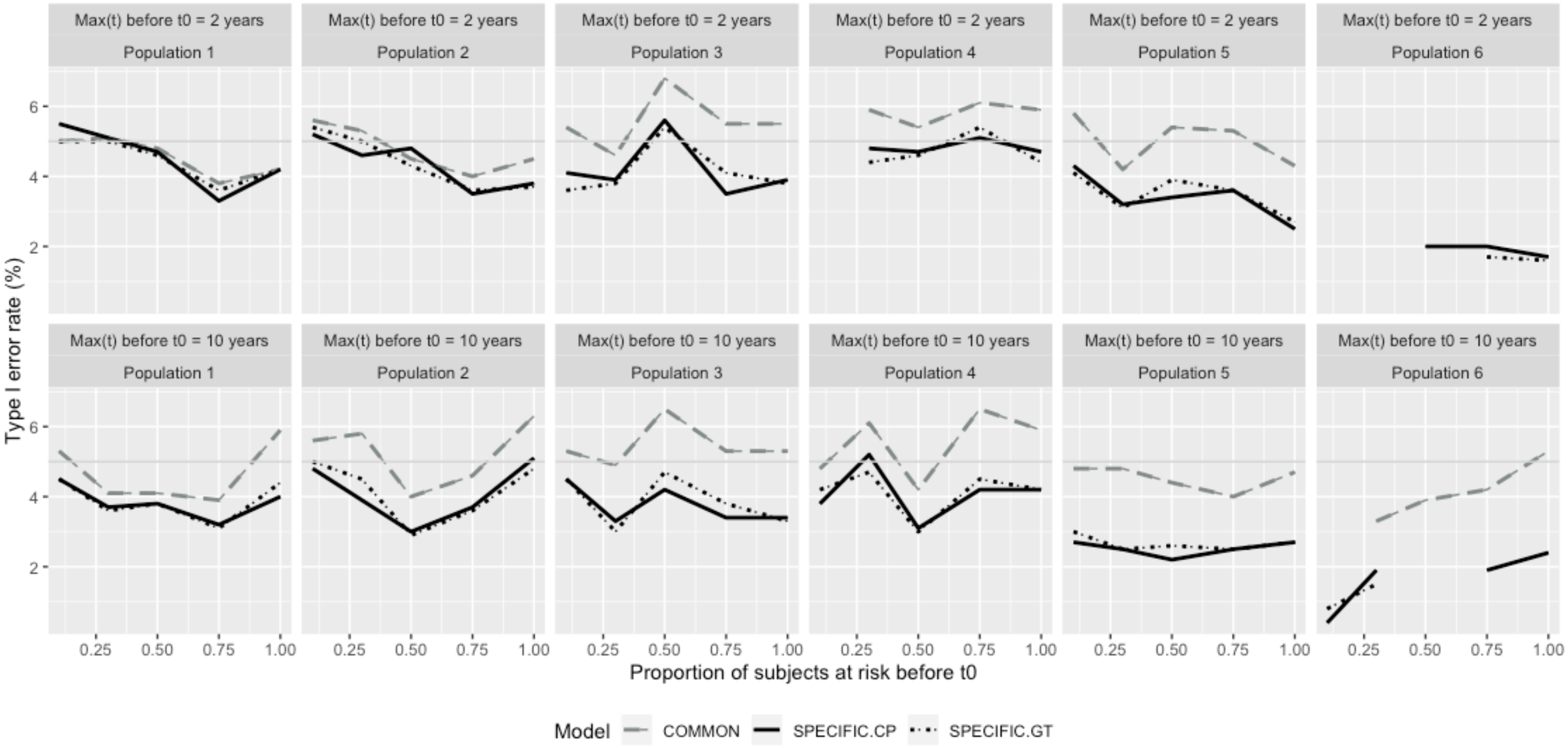

Supplement: Supplementary file 1 — Additional file 1. [file 12874_2022_1503_MOESM1_ESM.pdf]
