# Supplementary material for: Left-censored recurrent event analysis in epidemiological studies: a proposal for when the number of previous episodes is unknown
Source: BMC Med Res Methodol. 2022 Jan 16;22:20. doi: 10.1186/s12874-022-01503-1 (PMC8761288; doi:10.1186/s12874-022-01503-1)
Supplement: Supplementary file 2 — Additional file 2. [file 12874_2022_1503_MOESM2_ESM.docx]

**Table S1. Relative bias < 10%.**

| Subjects at risk before t=0 | Population 1 | | | | |  | Population 2 | | | | |  | Population 3 | | | | |  | Population 4 | | | | |  | Population 5 | | | | |  | Population 6 | | | | |
| --- | --- | --- | --- | --- | --- | --- | --- | --- | --- | --- | --- | --- | --- | --- | --- | --- | --- | --- | --- | --- | --- | --- | --- | --- | --- | --- | --- | --- | --- | --- | --- | --- | --- | --- | --- |
|  | 0.1 | 0.3 | 0.5 | 0.75 | 1 |  | 0.1 | 0.3 | 0.5 | 0.75 | 1 |  | 0.1 | 0.3 | 0.5 | 0.75 | 1 |  | 0.1 | 0.3 | 0.5 | 0.75 | 1 |  | 0.1 | 0.3 | 0.5 | 0.75 | 1 |  | 0.1 | 0.3 | 0.5 | 0.75 | 1 |
| *2 years max(t) before t=0* |  |  |  |  |  |  |  |  |  |  |  |  |  |  |  |  |  |  |  |  |  |  |  |  |  |  |  |  |  |  |  |  |  |  |  |
|  |  |  |  |  |  |  |  |  |  |  |  |  |  |  |  |  |  |  |  |  |  |  |  |  |  |  |  |  |  |  |  |  |  |  |  |
| COMMON | ✓ | ✓ | ✓ | ✓ | ✓ |  |  |  |  |  |  |  |  |  |  |  |  |  |  |  |  |  |  |  |  |  |  |  |  |  |  |  |  |  |  |
| SPECIFIC.CP | ✓ | ✓ | ✓ | ✓ | ✓ |  | ✓ | ✓ | ✓ |  |  |  | ✓ | ✓ |  |  |  |  | ✓ | ✓ | ✓ | ✓ |  |  | ✓ | ✓ | ✓ | ✓ |  |  | ✓ | ✓ | ✓ | ✓ | ✓ |
| SPECIFIC.GT | ✓ | ✓ | ✓ | ✓ | ✓ |  | ✓ | ✓ | ✓ | ✓ |  |  | ✓ | ✓ | ✓ | ✓ |  |  |  |  |  |  | ✓ |  |  |  |  | ✓ | ✓ |  |  | ✓ | ✓ | ✓ | ✓ |
| *5 years max(t) before t=0* |  |  |  |  |  |  |  |  |  |  |  |  |  |  |  |  |  |  |  |  |  |  |  |  |  |  |  |  |  |  |  |  |  |  |  |
|  |  |  |  |  |  |  |  |  |  |  |  |  |  |  |  |  |  |  |  |  |  |  |  |  |  |  |  |  |  |  |  |  |  |  |  |
| COMMON | ✓ | ✓ | ✓ | ✓ |  |  |  |  |  |  |  |  |  |  |  |  |  |  |  |  |  |  |  |  |  |  |  |  |  |  |  |  |  |  |  |
| SPECIFIC.CP | ✓ | ✓ | ✓ | ✓ | ✓ |  | ✓ | ✓ | ✓ |  |  |  | ✓ | ✓ |  |  |  |  | ✓ | ✓ | ✓ |  |  |  | ✓ | ✓ | ✓ | ✓ | ✓ |  | ✓ | ✓ | ✓ | ✓ | ✓ |
| SPECIFIC.GT | ✓ | ✓ | ✓ | ✓ | ✓ |  | ✓ | ✓ | ✓ |  |  |  | ✓ | ✓ | ✓ |  |  |  |  |  | ✓ | ✓ | ✓ |  |  | ✓ | ✓ | ✓ | ✓ |  |  | ✓ | ✓ | ✓ |  |

**Table S2. Coverage between 92.5% and 97.5%.**

| Subjects at risk before t=0 | Population 1 | | | | |  | Population 2 | | | | |  | Population 3 | | | | |  | Population 4 | | | | |  | Population 5 | | | | |  | Population 6 | | | | |
| --- | --- | --- | --- | --- | --- | --- | --- | --- | --- | --- | --- | --- | --- | --- | --- | --- | --- | --- | --- | --- | --- | --- | --- | --- | --- | --- | --- | --- | --- | --- | --- | --- | --- | --- | --- |
|  | 0.1 | 0.3 | 0.5 | 0.75 | 1 |  | 0.1 | 0.3 | 0.5 | 0.75 | 1 |  | 0.1 | 0.3 | 0.5 | 0.75 | 1 |  | 0.1 | 0.3 | 0.5 | 0.75 | 1 |  | 0.1 | 0.3 | 0.5 | 0.75 | 1 |  | 0.1 | 0.3 | 0.5 | 0.75 | 1 |
| *2 years max(t) before t=0* |  |  |  |  |  |  |  |  |  |  |  |  |  |  |  |  |  |  |  |  |  |  |  |  |  |  |  |  |  |  |  |  |  |  |  |
|  |  |  |  |  |  |  |  |  |  |  |  |  |  |  |  |  |  |  |  |  |  |  |  |  |  |  |  |  |  |  |  |  |  | ✓ | ✓ |
| COMMON | ✓ | ✓ |  |  |  |  |  |  |  |  |  |  |  |  |  |  |  |  |  |  |  |  |  |  |  |  |  |  |  |  | ✓ | ✓ | ✓ | ✓ | ✓ |
| SPECIFIC.CP | ✓ | ✓ | ✓ | ✓ | ✓ |  | ✓ | ✓ | ✓ |  |  |  | ✓ | ✓ |  |  |  |  | ✓ | ✓ | ✓ | ✓ |  |  | ✓ | ✓ | ✓ | ✓ | ✓ |  |  | ✓ | ✓ | ✓ | ✓ |
| SPECIFIC.GT | ✓ | ✓ | ✓ | ✓ | ✓ |  | ✓ | ✓ | ✓ |  |  |  | ✓ | ✓ | ✓ | ✓ |  |  |  |  |  |  |  |  |  |  |  | ✓ | ✓ |  |  |  |  |  |  |
| *5 years max(t) before t=0* |  |  |  |  |  |  |  |  |  |  |  |  |  |  |  |  |  |  |  |  |  |  |  |  |  |  |  |  |  |  |  |  |  |  |  |
|  |  |  |  |  |  |  |  |  |  |  |  |  |  |  |  |  |  |  |  |  |  |  |  |  |  |  |  |  |  |  |  |  |  |  |  |
| COMMON |  |  |  |  |  |  |  |  |  |  |  |  |  |  |  |  |  |  |  |  |  |  |  |  |  |  |  |  | ✓ |  |  | ✓ | ✓ | ✓ | ✓ |
| SPECIFIC.CP | ✓ | ✓ | ✓ | ✓ | ✓ |  | ✓ | ✓ | ✓ |  |  |  | ✓ |  |  |  |  |  | ✓ | ✓ | ✓ |  |  |  | ✓ | ✓ | ✓ | ✓ | ✓ |  | ✓ | ✓ |  | ✓ | ✓ |
| SPECIFIC.GT | ✓ | ✓ | ✓ | ✓ | ✓ |  | ✓ | ✓ | ✓ |  |  |  | ✓ | ✓ | ✓ |  |  |  |  |  |  |  | ✓ |  |  |  |  | ✓ | ✓ |  | ✓ | ✓ | ✓ | ✓ | ✓ |
